# Synthesis and Photophysical Properties of Electroactive Apocynin Derivatives as Potential Luminogens and Cellular Bioimaging Agents

**DOI:** 10.1021/acsomega.6c09229

**Published:** 2026-10-26

**Authors:** Camila García-Aquite, Luis Illicachi, Joel José Montalvo Acosta, Alejandro Ortiz, Homero G. San-Juan-Vergara, Juan D. Rodríguez, Walter A. Martínez-De-La-Rosa, Daniel Insuasty, Alberto Insuasty

**Affiliations:** † Grupo de Investigación de Compuestos Heterocíclicos, Departamento de Química, 28006 Universidad del Valle, Cali 760032, Colombia; ‡ Grupo de Investigación en Química y Biotecnología, Facultad de Ciencias Básicas, 28027 Universidad Santiago de Cali, Cali 760035, Colombia; § Facultad de Ciencias Farmacéuticas, 27986 Universidad de Cartagena, Cartagena 130015, Colombia; ∥ Departamento de Medicina, División Ciencias de la Salud, Universidad del Norte, Km 5 vía Puerto Colombia, Puerto Colombia 081007, Colombia; ⊥ Programa de medicina, Facultad de Ciencias de la Salud, Universidad Libre, Km 5 vía Puerto Colombia, Puerto Colombia 081007, Colombia; # Grupo de Investigación en Biomedicina, Departamento de Ciencias Básicas Medicas, Universidad Metropolitana, Barranquilla 080020, Colombia; ∇ Departamento de Química y Biología, División de Ciencias Básicas, Universidad del Norte, Puerto Colombia 081007, Colombia

## Abstract

This study describes the synthesis, photophysical characterization, and bioimaging properties of a series of chalcones derived from apocynin, functionalized with electroactive groups such as phenyldimethylamine (**1**), triphenylamine (**2**), tetraphenylethylene (**3**), and pyrene (**4**). These molecules featured donor−π–acceptor–donor (D−π–A–D) type architectures designed to promote intramolecular charge transfer (ICT) processes and notable emissive properties. Compounds **1** and **2** exhibited a marked aggregation-caused quenching (ACQ) behavior in THF/water mixtures while compounds **3** and **4** showed a marked enhancement of emission upon aggregation (AIE), associated with the restriction of intramolecular motions in the aggregated state. DFT/TD-DFT calculations and electrochemical studies revealed good electronic communications between the electroactive units. Cellular bioimaging studies performed on compounds **1–4** showed that only compounds **1**, **2**, and **4** are good fluorescent agents exhibiting notable cellular permeability in HEp-2 carcinoma cells. Overall, these findings demonstrate that apocynin α,β-unsaturated systems constitute versatile luminescent platforms with tunable photophysical properties, suitable for applications in cellular bioimaging and the development of remarkable fluorescent materials.

## Graphical abstract

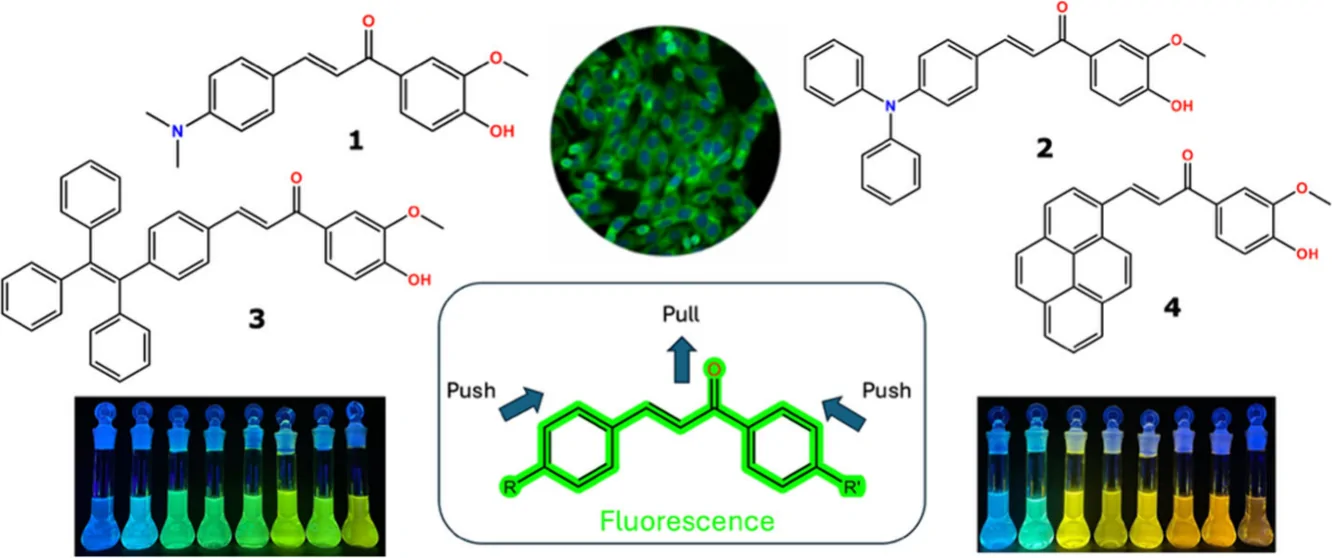

## 1 Introduction

Chalcones constitute a family of α,β-unsaturated compounds that have been extensively studied due to their natural abundance as precursors of flavonoids and isoflavonoids in plants.^1–4^ Numerous studies have demonstrated that chalcones exhibit relevant biological activities—including anticancer, anti-inflammatory, antioxidant, and antimicrobial effects.^5–9^ Moreover, chalcones are interesting because of their synthetic accessibility, which allows modulating electronic properties through the selective incorporation of electron-donating and electron-withdrawing groups into donor−π–acceptor–donor (D−π–A–D) architectures producing fluorescent molecules suitable for bioimaging purposes (Figure 1) —which reinforces their potential as functional biomarkers in biological environments.^3,4,10^ This structural flexibility enables the design of molecules with efficient intramolecular charge transfer (ICT), large Stokes shifts, high microenvironmental sensitivity, and the ability to generate intense optical signals within complex biological systems.^11,12^

**Figure 1:**
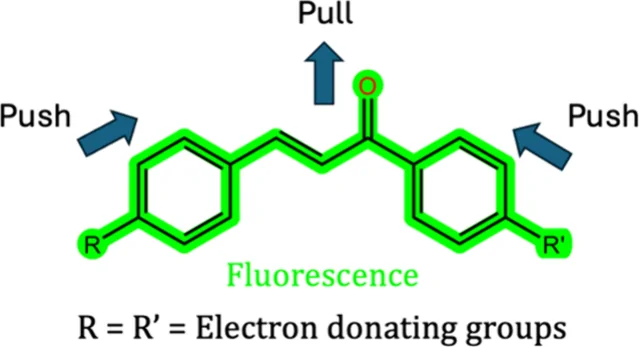
Structural design of fluorescent chalcones.

In recent years, several studies have shown that chalcones can act as fluorescent probes sensitive to variations in polarity, viscosity, aggregation, oxidative stress, and enzymatic activity, all of which are highly relevant features within the tumor microenvironment.^11,13^ Their ability to electronically reorganize the π-conjugated system in response to altered intracellular conditions makes them excellent biomarkers for the detection and visualization of living cancer cells.^12^ Likewise, the incorporation of strong donor groups such as phenyldimethylamine or triphenylamine (*N*-based), and C-based units such as tetraphenylethylene (TPE) and pyrene, enhances emission intensity, suppresses nonradiative deactivation, and promotes, in certain cases, aggregation-induced emission (AIE), a particularly useful property in cellular bioimaging or high-density molecular regions such as tumor tissues.^14,15^

Aggregation-induced emission (AIE) has emerged as an important principle in the design of new fluorophores for biological applications.^16^ Unlike conventional fluorophores, which usually undergo aggregation-caused quenching (ACQ) behavior, AIEgen molecules enhance their emission in ordered or aggregated states due to the restriction of intramolecular rotations and vibrations.^17,18^ Among them, tetraphenylethylene (TPE) stands out as one of the most versatile and efficient AIE luminogens. Its highly twisted architecture acts as a molecular rotor, rendering the molecules nearly nonemissive in solution but strongly fluorescent in aggregated states.^19,20^ These effects, combined with the photochemical stability of the TPE core, have led to the development of systems with applications in mechanical sensors, optical data storage, analyte detection, and smart platforms exhibiting reversible mechanochromic behavior.^21,22^ Similar systems exhibiting AIE properties based on triphenylamine or pyrene moieties for biological applications have also been reported.^23,24^

The chalcone framework also allows the incorporation of bioactive phenolic nuclei such as apocynin, which provides electronic stabilization through its donor groups and enables the development of hybrid molecules with biological relevance and tunable photophysical behavior.^12^ Chalcones derived from apocynin, along with their functionalization with other electroactive units such as pyrene, triphenylamine, or phenyldimethylamine moieties, exemplify rigid or semirigid systems capable of controlling torsion around the π-acceptor bridge, thereby modulating ICT processes, quantum yield, and photochemical stability.^25–27^

Experimental characterization of these systems—including absorption and emission studies, quantum-yield determination, lifetime measurements, and solvent-dependent behavior—combined with theoretical approaches (DFT/TD-DFT) enables the correlation of electronic parameters such as HOMO–LUMO energies, chemical potential, reactivity descriptors, and atomic charge distribution with the observed optical properties.^28,29^ In this context, the apocynin chalcones synthesized in this work constitute a highly adaptable molecular platform capable of generating not only fluorescent systems but also possible anticancer agents, whose optical properties can be modulated, making them suitable scaffolds for bioimaging and selective detection of cancer cells. Altogether, these features position them as promising candidates for applications in diagnosis and image-assisted therapy.

Herein, we propose the synthesis of apocynin-based fluorescent systems functionalized with different electroactive units such as phenyldimethylamine (**1**), triphenylamine (**2**), tetraphenylethylene (**3**), and pyrene (**4**) moieties. Their optical and photophysical properties have been well-studied as well as their behavior as AIEgens and as cellular bioimaging agents.

## 2 Results and Discussion

### 2.1 Apocynin-Based Systems

#### 2.1.1 Synthesis

The synthesis of the fluorescent chalcones **1–4** is shown in Scheme 1. All molecules were prepared through a Claisen–Schmidt condensation between 1-(4-hydroxy-3-methoxyphenyl)­ethan-1-one (Apocynin) and a series of structurally diverse aromatic aldehydes. These aldehydes include strong and moderate electron-donating groups such as phenyldimethylamine, triphenylamine, tetraphenylethylene, and pyrene, allowing the yield of chalcones **1**, **2**, **3**, and **4** (respectively) in donor−π–acceptor–donor (D−π–A–D) architectures (Scheme 1). The reaction was carried out in methanol using KOH as base at room temperature, followed by neutralization with acetic acid and separation of the resulting precipitate by filtration. The desired compound was purified by column chromatography on silica gel using dichloromethane as eluent to afford compounds **1–4** with yields of 78–91% (see Experimental Section). Spectroscopical data such as ^1^H and ^13^C NMR, mass spectrometry, and IR of target compounds (**1–4**) can be found in the Supporting Information (Figures S1–S12).

**Scheme 1:**
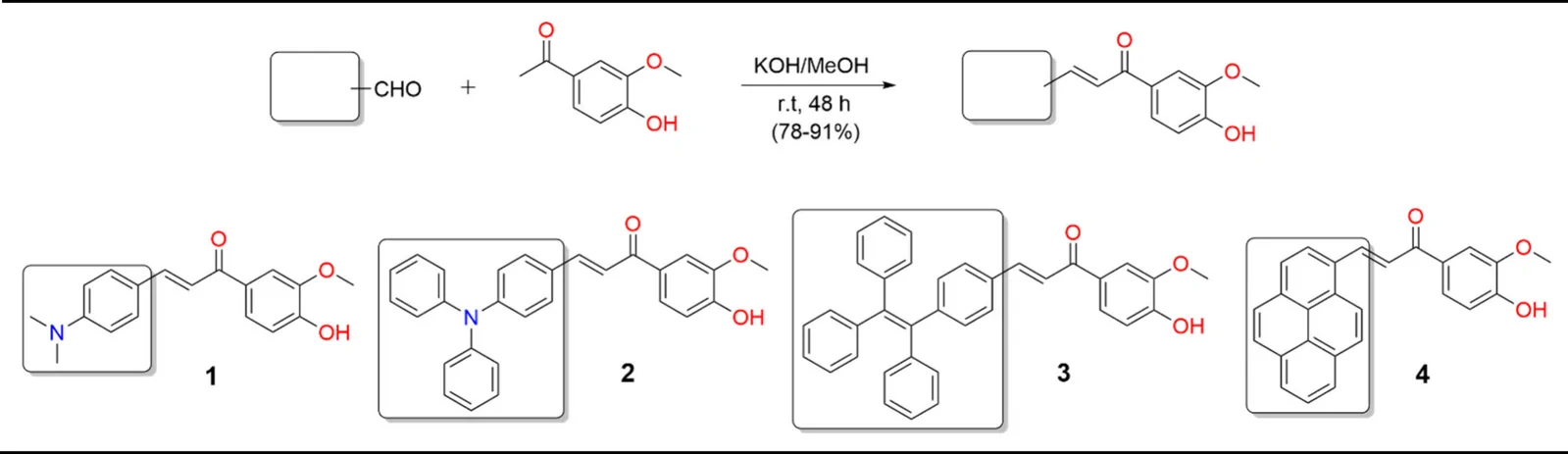
Synthesis of Chalcones Derived from Apocynin **1**–**4**

#### 2.1.2 Photophysical Properties

The optical properties of compounds **1–4** were determined by UV–Vis and fluorescence emission studies in eight different solvents with increasing polarity (toluene, THF, DCM, acetone, DMF, DMSO, ACN, and EtOH), as outlined in Table 1. The absorption spectra of compounds **1–4** are shown in Figure 2, where compounds **1** and **2**, bearing strong nitrogen-based electron-donor groups (phenyldimethylamine and triphenylamine, respectively), exhibited maximum absorption bands in the range of 398–417 nm, which are assigned to an intramolecular charge transfer (ICT) transition from the electron-rich group to the electron-deficient carbonyl group.^28^ In contrast, for compound **3**, bearing a tetraphenylethylene unit, absorption maxima are located at shorter wavelengths in the range of 340–390 nm, which is assigned to π–π* transitions along with ICT processes due to the push–pull effect of a weaker electron donor unit such as TPE in comparison to *N*-based donor units in **1** and **2**.^30^ For compound **4**, bearing a pyrene unit, broad absorption spectra were observed in all solvents, with absorption maxima in the range of 350–380 nm assigned to π–π* transitions featuring a notable shoulder at longer wavelengths in the range of 400–430 nm attributed to ICT transitions from the pyrene unit to the carbonyl group. The π–π* transition band for compound **4** is clearly red-shifted in comparison to compound **3** due to a more π conjugated planar system when pyrene is compared to TPE, favoring the electronic communication between the electroactive units and exhibiting a more pronounced red-shifted ICT band as well. Regarding molar absorption, the ε values for compounds **1–4** are similar to those obtained for related electroactive apocynin derivatives ranging from 1.6 × 10^4^ up to 5.5 × 10^4^ L·mol^–1^·cm^–1^.^12^

**Table 1:** Spectroscopic Properties of Compounds **1**–**4**

Compound	**1**			**2**			**3**			**4**		
Solvent	Abs, nm (ε, M^–1^cm^–1^)	Em, nm (φ)^a^	Stokes shift, cm^–1^ (nm)	Abs, nm (ε, M^–1^cm^–1^)	Em, nm (φ)^a^	Stokes shift, cm^–1^ (nm)	Abs, nm (ε, M^–1^cm^–1^)	Em, nm (φ)^a^	Stokes shift, cm^–1^ (nm)	Abs, nm (ε, M^–1^cm^–1^)	Em, nm (φ)^a^	Stokes shift, cm^–1^ (nm)
Toluene	400 (23377)	462 (0.019)	3355 (62)	407(25789)	478 (0.013)	3650 (71)	363 (21171)	472 (0.002)	6362 (109)	373 (21203)	460 (0.015)	5071 (87)
THF	398 (40583)	479 (0.013)	4249 (81)	401 (51265)	507 (0.034)	5214 (106)	360 (45275)	507 (0.004)	8054 (147)	386 (32598)	470 (0.003)	4630 (84)
DCM	411 (29271)	507 (0.004)	4607 (96)	415 (55371)	542 (0.012)	5646 (127)	361 (31607)	532 (0.01)	8904 (171)	365 (37514)	495 (0.019)	7195 (130)
Acetone	401 (34194)	512 (0.025)	5406 (111)	403 (38099)	538 (0.018)	6227 (135)	360 (33296)	522 (0.002)	8621 (162)	383 (49025)	485 (0.006)	5491 (102)
DMF	410 (28233)	517 (0.048)	5048 (107)	408 (50094)	541 (0.019)	6026 (133)	362 (24183)	520 (0.004)	8394 (158)	388 (24473)	492 (0.007)	5448 (104)
DMSO	417 (25950)	529 (0.013)	5077 (112)	414 (33119)	550 (0.003)	5973 (136)	367 (24770)	534 (0.001)	8521 (167)	390 (21119)	504 (0.003)	5800 (114)
ACN	406 (15555)	522 (0.064)	5473 (116)	405 (41610)	551 (0.006)	6543 (146)	355 (32532)	534 (0.003)	9442 (179)	363 (25493)	505 (0.016)	7746 (142)
EtOH	415 (38166)	537 (0.017)	5474 (122)	415(28263)	546 (0.004)	5781 (131)	369 (30096)	538 (0.039)	8513 (169)	366 (23324)	532 (0.029)	8525 (166)

a Relative quantum yield using Prodan as a standard.

**Figure 2:**
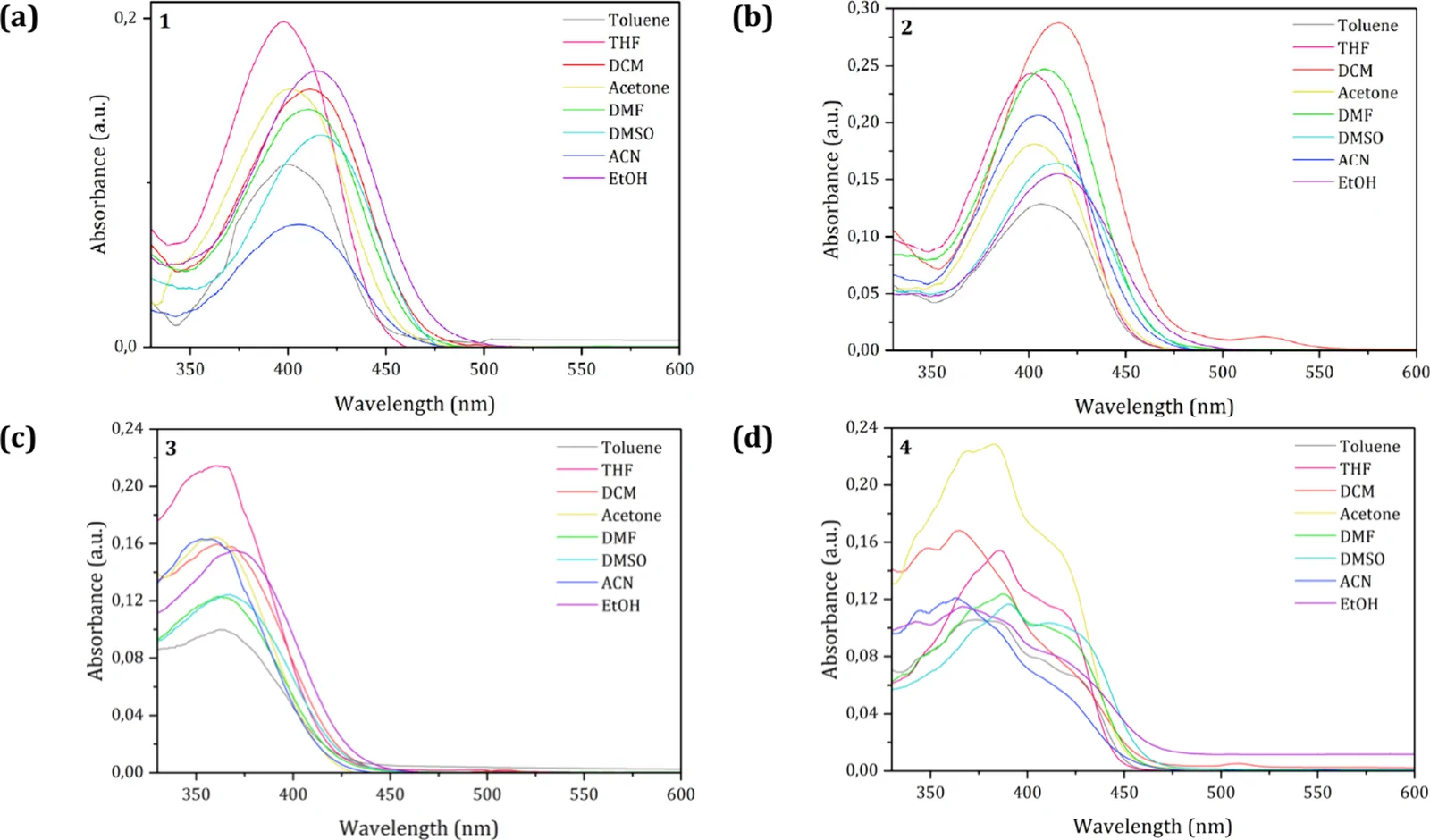
Absorption spectra of **1** (a), **2** (b), **3** (c), and **4** (d) (5.0 μM) in different solvents.

In all cases, the absorption wavelengths exhibited slight solvent-dependent shifts as can be observed in Figure 2 and Figure S13; however, the emission bands showed a much more pronounced variation, reaching shifts at wavelengths between 460 and 551 nm (Figure 3). Interestingly, these compounds exhibited large Stokes shifts (**1**, 3355–4474; **2**, 3650–6543; **3**, 6362–9442; **4**, 4630–8525 cm^–1^) in different solvents (Table 1), which is beneficial for fluorescence imaging as it maximizes signal-to-noise ratios by separating detection from scattered excitation light, as well as favoring the minimization of undesired self-absorption or autofluorescence processes.^31^ In all cases, a tendency is observed in which an increase in the polarity of the solvent leads to an increase in the Stokes shift value. These observations suggest that the excited state exhibits a markedly higher polar character than the ground state, resulting in a stronger interaction with polar solvents.^32^ Regarding the fluorescence quantum yields (φ) (Table 1), for compounds **1** and **2**, bearing strong electron-donor units, it was observed that when the polarity of the solvent increases from nonpolar to medium-polar ones, the φ generally exhibited an enhancement; this could be due to the stabilization of a highly emissive, planar intramolecular charge transfer (ICT) state, reducing the nonradiative decay rates.^33^ In highly polar solvents, the quantum yield decreases, probably due to the formation of dark twisted intramolecular charge transfer (TICT) states.^34^ For compounds **3** and **4**, bearing weak to moderate electron-donor units, the quantum yield values were found to be, generally, lower than that of compounds **1** and **2**, attributed to less efficient ICT processes for **3** and **4**.^35,36^ In highly polar solvents, compounds **3** and **4**, increased their φ values, assigned to the stabilization of the intramolecular charge transfer state.

**Figure 3:**
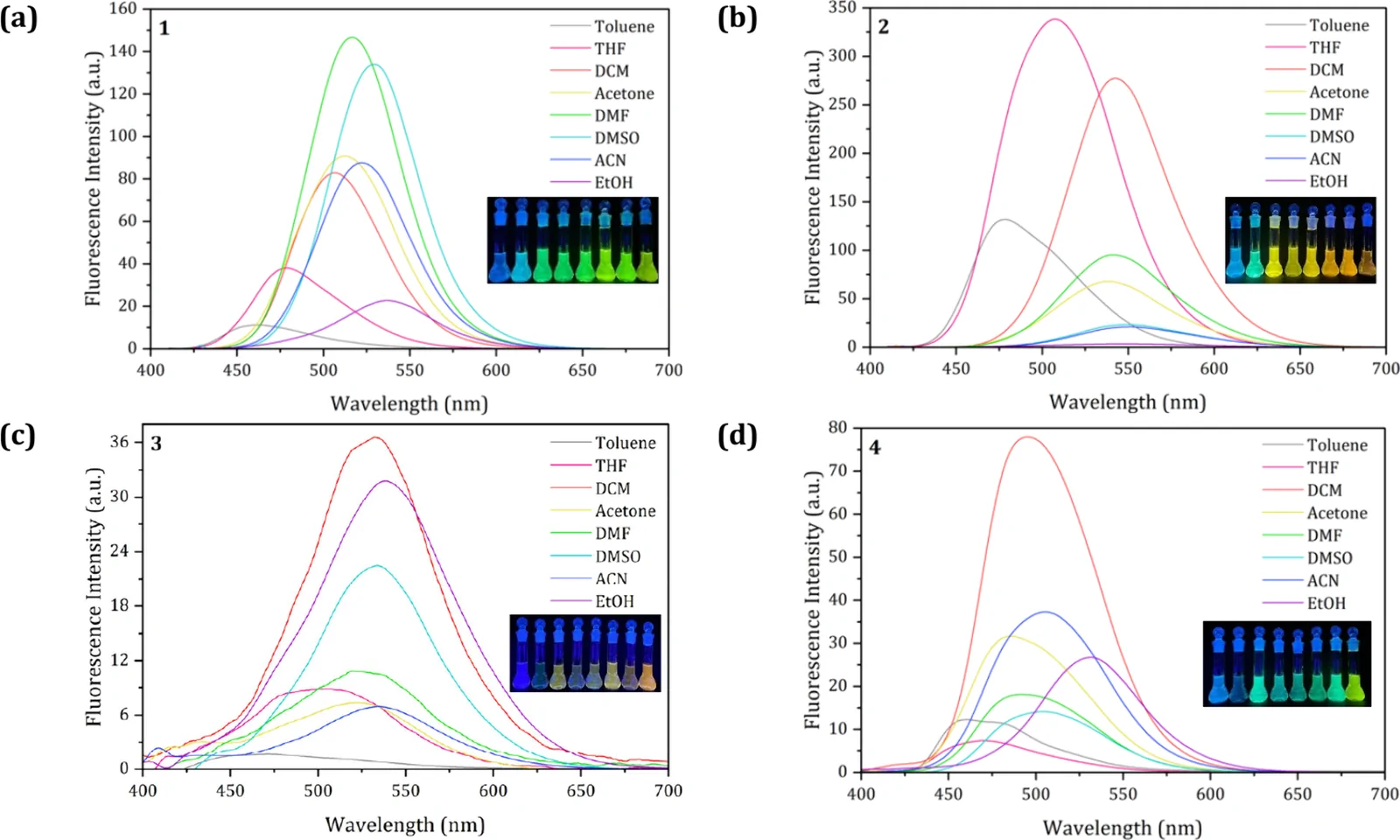
Emission spectra of **1** (a), **2** (b), **3** (c), and **4** (d) (5.0 μM) in different solvents. Insets correspond to pictures of solutions under UV light (365 nm lamp) in solvents with increasing polarity.

#### 2.1.3 Solvatofluorochromism

The marked differences in the Stokes shifts observed for compounds **1–4** motivated a more detailed study of the emission properties of fluorophores **1–4** in solvents of different polarity. All compounds exhibited color changes from blue in solvents of low polarity (toluene and THF) to green (for **1** and **4**) or orange (for **2** and **3**) in a highly polar solvents (EtOH) (Figure 4a–d). The study of the emission spectra revealed a shift toward longer wavelengths (bathochromic) as the polarity of the solvent increased, that is, a positive solvatochromic behavior, occurring because the molecule’s excited state is more polar and stabilized by a polar solvent.^37,38^

**Figure 4:**
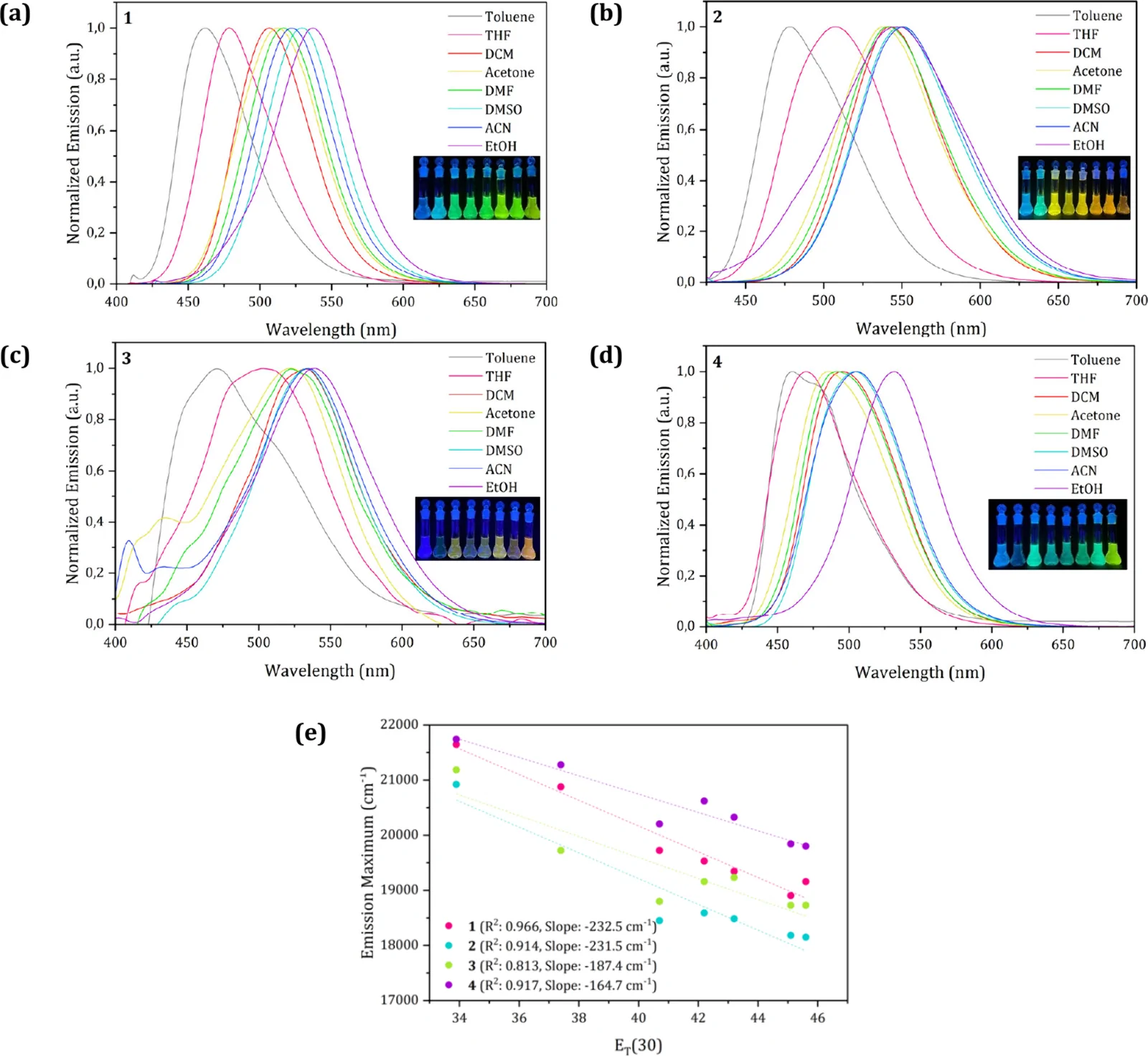
Normalized emission spectra of **1** (a), **2** (b), **3** (c), and **4** (d) (5.0 μM) in different solvents at 25 °C. Insets are pictures of solutions under UV light (365 nm lamp) in solvents with increasing polarity. (e) Plot of emission maximum vs E_T_ (30) in aprotic solvents.

To quantitatively describe this effect, different correlation approaches with solvent polarity were employed, including the dependence of the emission maximum on the empirical polarity scale E_T_(30) (Figure 4e), which shows a clear linear trend with negative slopes for all compounds, confirming a pronounced positive solvatochromism.^39^ The obtained slopes indicate a significant variation of the dipole moment between the ground and excited states, −232.5 cm^–1^ (*R* ^2^ = 0.97), −231.5 cm^–1^ (*R* ^2^ = 0.91), −187.4 cm^–1^ (*R* ^2^ = 0.81), and −164.7 cm^–1^ (*R* ^2^ = 0.92) for compounds **1**, **2**, **3** and **4**, respectively, evidencing a strong charge-transfer character in the excited state. The high *R* ^2^ values suggest that solvent polarity is the main factor governing the emission, in agreement with the preferential stabilization of the ICT state in polar media.^40^

Similarly, the Stokes shifts were analyzed as a function of the orientational polarizability of the medium (Δ*f*) using the Lippert–Mataga relationship (see Figure S14a in the Supporting Information), where positive trends were observed for compounds **1**, **2**, and **3**. This confirms a significant change in dipole moment between the ground and excited states, consistent with the formation of intramolecular charge transfer (ICT) states. Likewise, the linear correlation observed in the Rettig plots (Figure S14b) suggests that compounds **1** and **2** (*R* ^2^ = 0.92 and 0.98, respectively) exhibit TICT processes caused by their flexible donor *N*,*N*-dimethylamine and triphenylamine groups. The stabilization of the dark TICT state facilitates rapid nonradiative relaxation to the ground state, resulting in low fluorescence quantum yield in more polar solvents.^41^ Nevertheless, the poor linear correlation obtained for compound **4** in both the Lippert–Mataga and Rettig plots can be attributed to the presence of the pyrene group, which introduces additional localized excited states and promotes π–π interactions. Due to the high tendency of pyrene to form aggregates and excimers, the emission of compound **4** may originate not only from ICT states but also from locally excited (LE) states, whose stabilization strongly depends on supramolecular organization rather than solely on solvent polarity. Consequently, the Stokes shift does not follow a linear trend with Δ*f*, evidencing a more complex excited-state relaxation process.^42^

#### 2.1.4 Aggregation Properties

To evaluate the influence of aggregation on the photophysical behavior of compounds **1**–**4**, emission spectra were recorded in THF/water mixtures with increasing water fractions (*f*_W_ = 0–95%), as shown in Figure 5. In all cases, increasing the water content produced significant changes in both the emission intensity and the position of the emission maxima, evidencing a marked sensitivity to the surrounding medium. In all cases, increasing the water content produced significant changes in both the emission intensity and the position of the emission maxima, evidencing a marked sensitivity to the surrounding medium, which is driven by two distinct, and sometimes competing, contributions: the increase of solvent polarity and the physical aggregation of the fluorophores due to the reduced solubility. For compounds **1** and **2**, a progressive decrease in fluorescence intensity was observed with increasing *f*_W_, attributable to their aggregation. At a low-to-moderate water fraction, the increase in the medium’s macroscopic polarity stabilizes a highly polar excited state. This leads to positive solvatofluorochromism and facilitates the formation of a nonradiative TICT dark state, thereby depleting the excitation energy. As the *f*_w_ increases, reduced solubility triggers the formation of nanoaggregates. In this aggregate state, π-staking creates a new nonradiative trap state, forcing the molecules into an aggregation-caused quenching (ACQ) state.^37^ It is worth noting that for compound **2**, a dramatic decrease of fluorescence is observed at only 10% *f*_W_, while for compound **1**, the decrease of fluorescence is progressive as the *f*_W_ increases (Figure 5a,b). This fact could be attributed to a lower solubility of compound **2** in aqueous media, due to the triphenylamine moiety, which triggers a rapid aggregation of **2** compared to **1**.

**Figure 5:**
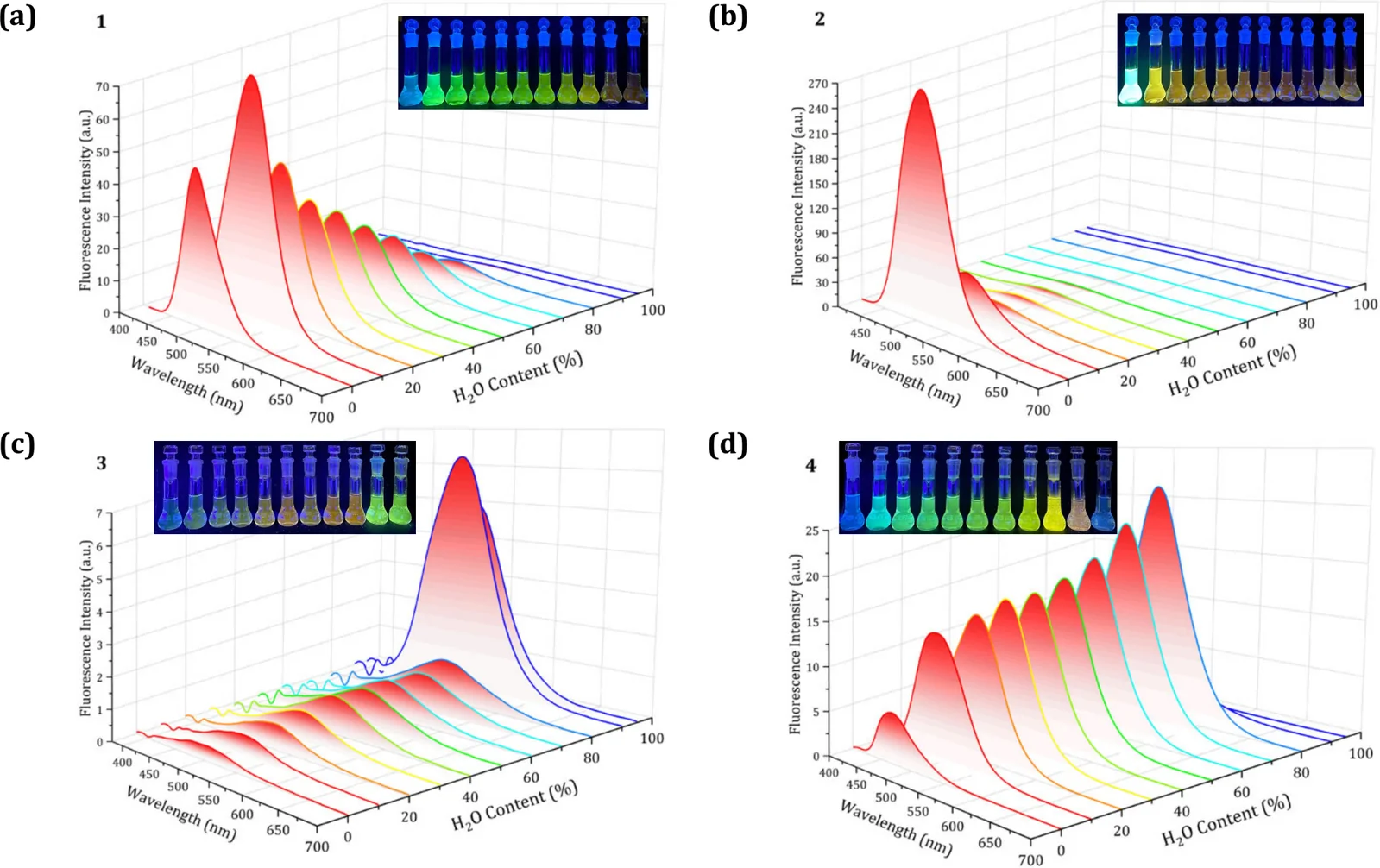
Emission spectra of **1** (a), **2** (b), **3** (c), and **4** (d) (5 mM) in THF/water mixtures.

In contrast, compounds **3** and **4** exhibited an increase in emission intensity as the water content increased (Figure 5c,d). The most notable case corresponds to compound **3**, for which a maxima fluorescence intensity is achieved at 90% *f*_W_ with a 15-fold increased fluorescence regarding the initial emission intensity (0% *f*_W_), this behavior suggests an aggregation-induced emission (AIE) effect, attributed to the restriction of intramolecular motions (RIM) mechanisms, in the aggregated state (triggered by a high water content). Particularly, the restriction of phenyl ring rotations associated with the tetraphenylethylene unit effectively suppresses nonradiative deactivation pathways. Compound **4** also showed an AIE behavior; however, the increase in fluorescence intensity, which in this case is less notable in comparison to **3**, is observed at lower *f*_W_ values and reaches a maximum at 80% *f*_W_ with a 5-fold increased fluorescence regarding the initial emission intensity (0% *f*_W_), followed by a dramatic quenching of fluorescence. This result probably indicates the coexistence of emissive aggregation processes caused by restricted intramolecular motions and partial self-quenching, possibly associated with π–π interactions between aromatic units, especially those of the pyrene fragment.^43–45^ Interestingly, it is observed that *N*-based (**1** and **2**) and *C*-based (**3** and **4**) apocynin systems give rise to different fluorescence aggregation behaviors, ACQ and AIE, respectively. We believe these main facts can be considered: It was observed that *N*-based compounds (**1** and **2**) can undergo TICT processes, which depletes the excitation energy before it can fluoresce. Moreover, in the aggregated state, the dense π-stacking for **1** and **2**, could create new nonradiative trap states, which drain the excited state energy and force the molecule into an ACQ state.^46^

#### 2.1.5 Viscosity Studies

The development of sensitive fluorescent probes allowing the study of local microviscosity domains in biological systems and materials is highly desirable. It is well-known that viscosity is a critical parameter in the cellular microenvironments, influencing molecular transport, diffusion of metabolites, and interactions between biomolecules.^47,48^ In this regard, viscosity studies were performed by measuring the emission spectra of compounds **1**–**4** in EtOH/glycerol mixtures with increasing glycerol content (0–95%). This study allowed a progressive increase of viscosity in the medium without introducing drastic changes in polarity, making it suitable for studying photophysical processes governed by intramolecular motions in the excited state.^13^ For all compounds, a progressive increase in fluorescence intensity was observed as the glycerol content increased, evidencing a strong dependence of emission on the medium’s viscosity, as shown in Figure 6. This behavior suggests that the restriction of intramolecular rotation (RIR) mechanism plays a key role in suppressing the nonradiative deactivation pathways and activating the release of energy through the fluorescence channels in the excited state.^29,49^

**Figure 6:**
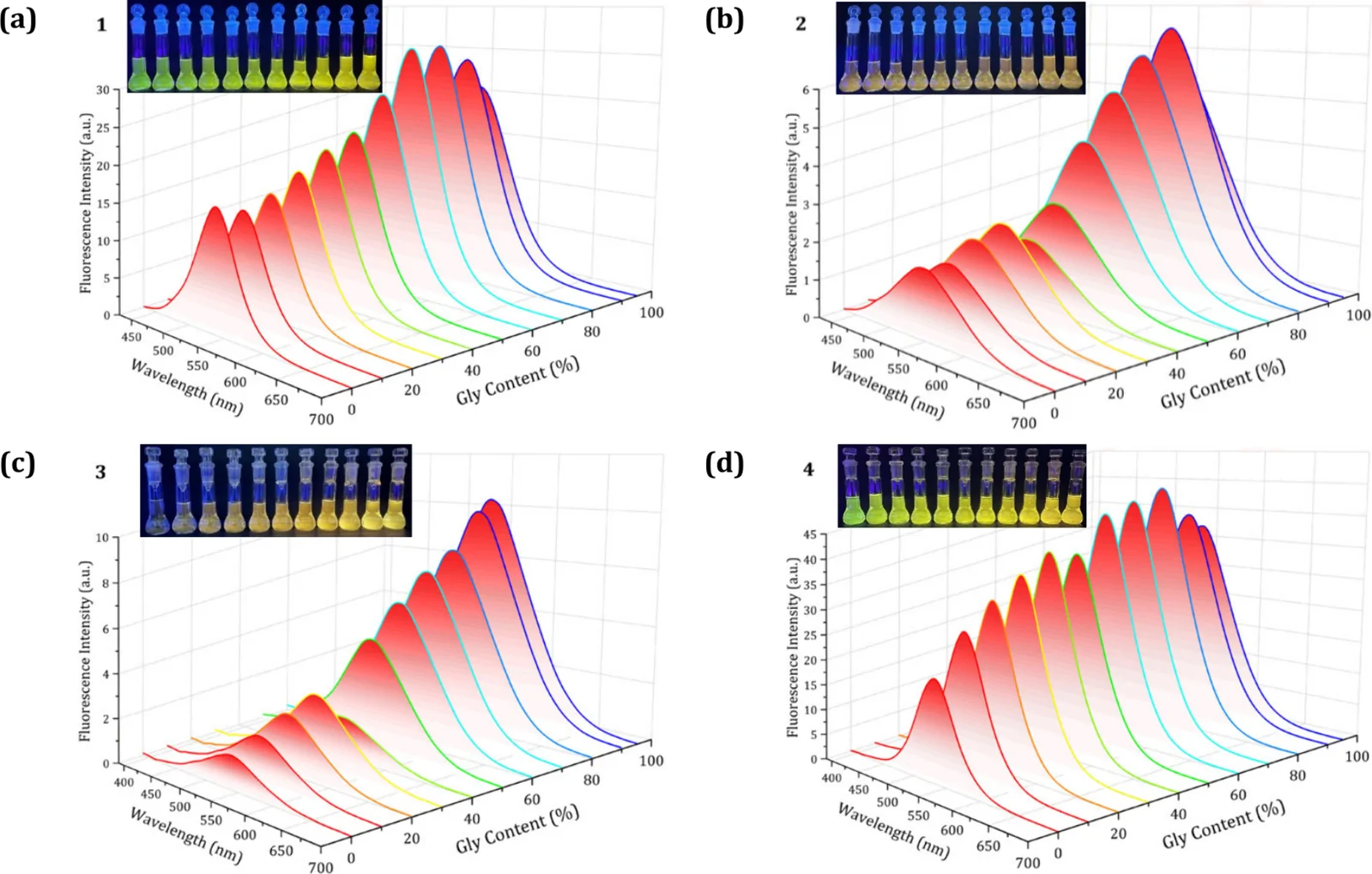
Emission spectra of **1** (a), **2** (b), **3** (c), and **4** (d) (5 mM) in EtOH/Glycerol mixtures.

All compounds **1**–**4** exhibited a gradual and continuous increase in emission intensity with increasing glycerol fractions (Figure 6). This behavior is consistent with fluorophores dominated by intramolecular charge transfer (ICT) processes, in which increased viscosity restricts the torsional motions of the donor groups bonded to the ethylene bridge, reducing the efficiency of nonradiative channels and favoring radiative emissions. Compounds **2** and **3** showed the most pronounced increase in fluorescence intensity across the entire EtOH/glycerol composition range, increasing their fluorescence more than 5- and 10-fold regarding the initial emission intensity (Figure 6b,c). This result reflects a strong sensitivity to viscosity, attributable to the restriction of intramolecular motions of all phenyl rotors in **2** and **3** induced by the viscous medium, triphenylamine, and tetraphenylethylene units, respectively. The effective suppression of intramolecular motions favors the radiative deactivation of the excited state, leading to an amplified emissive response.^50^ This behavior confirms the potential of these fluorophores as probes sensitive to viscous microenvironments with possible applications in bioimaging and in monitoring local viscosity changes in complex systems.

#### 2.1.6 Computational Calculations

The electronic and excited states properties of the synthesized chalcone derivatives were investigated by chemical quantum calculations using ωB97X-D3­(BJ)/def2-TZVP. In the first place, the energies for the highest occupied molecular orbital (*E*_HOMO_), the lowest unoccupied molecular orbital (*E*_LUMO_), and the HOMO–LUMO energy gap, were computed for the four chalcone derivatives **1**–**4** from ground-state optimized geometries at the DFT level in vacuum, as shown in Table S1 (see the Supporting Information). Theoretical calculations revealed that the first excited states (S_1_), responsible for the most intense absorption bands, exhibit high transition intensities, as reflected by their oscillator strengths (*f* = 1.11, 1.16, 1.01, and 0.94 for compounds **1**, **2**, **3**, and **4**, respectively).^32^ These values indicate highly allowed electronic transitions and confirm that the S_0_ → S_1_ processes are dominated by π → π* transitions, consistent with the strong experimental absorption in the near-UV region. Likewise, the HOMO energies for compounds **1**, **2**, **3**, and **4** fall within a narrow range (−7.50 to −7.90 eV), reflecting their structural similarity and the shared apocynin scaffold. However, the most relevant differences appear in their LUMO levels: compounds **3** (−0.11 eV) and **4** (−0.43 eV) show significant LUMO stabilization relative to **1** (0.33 eV) and **2** (0.079 eV), indicating a reduction in the HOMO–LUMO gap. This explains why compounds **3** and **4** exhibit larger Stokes shifts and the stronger solvatofluorochromic behavior observed experimentally.^31,40^

Complementary visualization of the HOMO and LUMO orbitals are shown in Figure 7. For all compounds **1**–**4**, the HOMO orbital density is predominantly localized on the electron-donating group and the conjugated core, which is typical for π → π* transitions in donor–acceptor systems. For all four compounds, the LUMO is largely delocalized across the conjugated core including the phenyl ring linked to the ethenyl group and extends significantly onto the electron-withdrawing group (α,β-unsaturated system with the carbonyl group), which acts as the π* acceptor part of the transition. Next, TD-DFT was used to compute the first three singlet excited-state transitions for the compounds in implicit THF solvent. The key results, including the vertical excitation energy, corresponding wavelength, and oscillator strength are presented in Table 2. In order to elucidate the electronic nature of the excitations, we evaluated the dominant natural transition orbitals^51^ (NTOs) for the first three excited states of each compound (see Table 2). Across all four molecules, the first excited state (S_1_), responsible for the most intense absorption band, is dominated by single-orbital transitions with pronounced HOMO → LUMO character (S_0_ → S_1_). This is quantitatively confirmed by the NTO analysis, which reveals single NTO pairs accounting for 70–97% of the transition weights across nearly all four chalcones. Such high single-pair contributions denote electronically simple excitations with limited multiconfigurational mixing, making the photophysics straightforward to interpret. Exceptionally, **3** and **4** display more orbital mixing in the S_2_–S_3_ states through contributions from HOMO(−1) and LUMO­(+1), in addition to HOMO–LUMO contributions, leading to a more complex NTO pattern. The combination of high oscillator strengths (especially for S_1_) and strong HOMO → LUMO NTO contributions indicates that the first three excitations correspond almost exclusively to π → π* transitions. Finally, a linear correlation (*R* ^2^ = 0.81) between the predicted vertical excitation energies and the ground-state HOMO–LUMO gaps is found.

**Table 2:** Computed Vertical Excitation Energies, Corresponding Wavelength, Oscillator Strength, and the Nature of the Transition (from NTO Analysis) for the First Three Excited States for the Four Chalcones **1**–**4** Assessed from TD-DFT in THF

Compound	Excited state	Transition	% Contribution (from NTO)	Excitation energy (eV)	Wavelength (nm)	Oscillator strength
**1**	1	HOMO–LUMO	89.26	3.95	313.92	1.1083
	2	HOMO–LUMO	96.27	4.18	297.01	0.0452
	3	HOMO–LUMO	69.70	4.65	266.55	0.0577
**2**	1	HOMO–LUMO	85.38	3.81	325.29	1.1630
	2	HOMO–LUMO	97.15	4.17	297.50	0.0193
	3	HOMO–LUMO	71.26	4.43	279.97	0.0394
**3**	1	HOMO–LUMO	74.60	3.97	312.42	1.0959
	2	HOMO–LUMO	93.54	4.14	299.59	0.0461
	3	HOMO–LUMO	27.67	4.56	271.99	0.3092
		HOMO(−1)–LUMO(+1)	14.43			
**4**	1	HOMO–LUMO	83.44	3.71	333.87	0.9449
	2	HOMO–LUMO	27.27	4.02	308.33	0.0093
		HOMO(−1)–LUMO(+1)	19.92			
	3	HOMO–LUMO	92.24	4.13	299.99	0.0091

**Figure 7:**
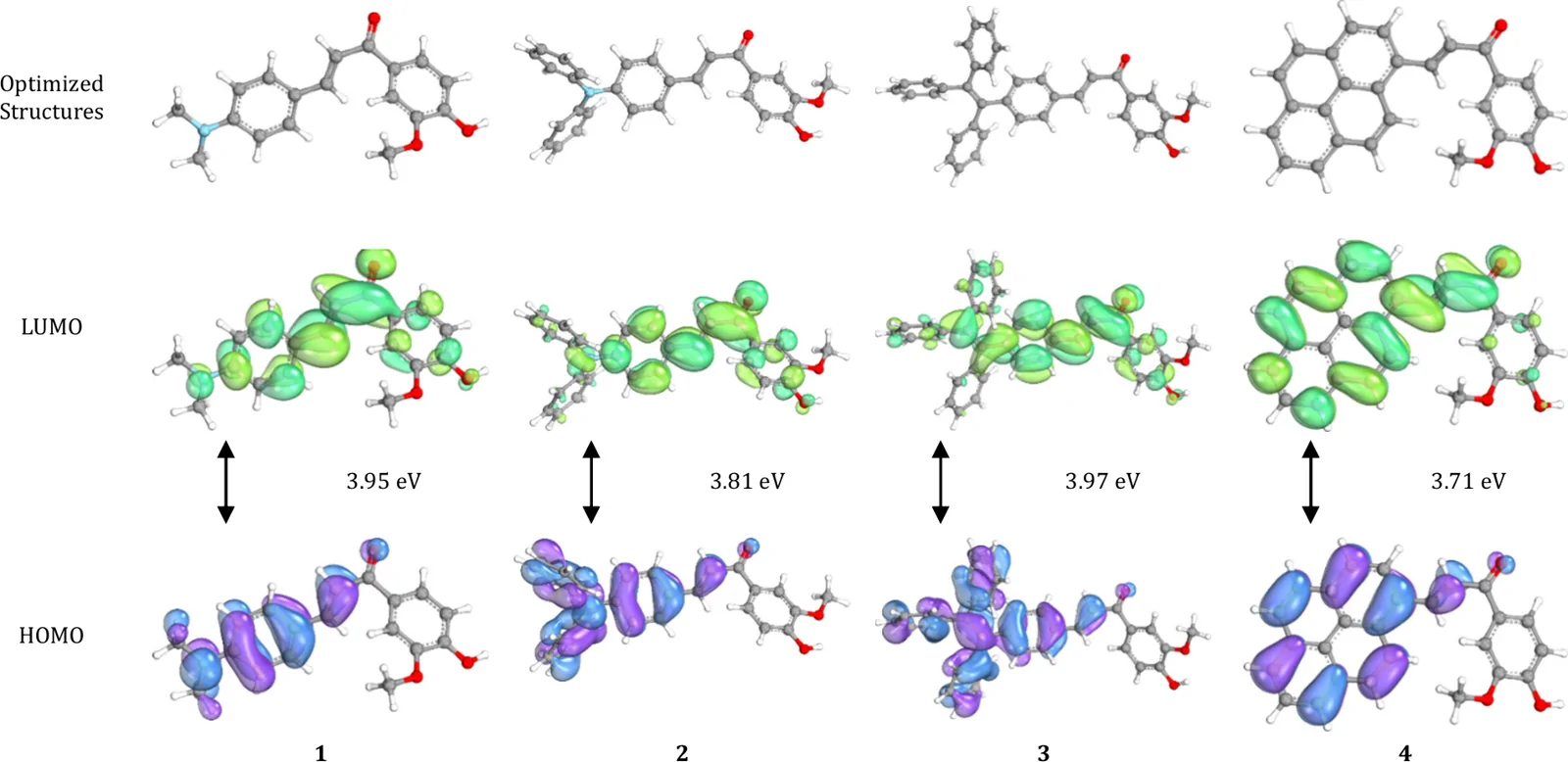
Optimized structures, HOMO and LUMO representations for compounds **1**–**4**.

Additionally, as a representative example, the dipole moments of compound **1** were extracted from the final converged values for three different calculations: (i) geometry optimization in the ground state (S_0_) at THF and EtOH, (ii) vertical TD-DFT single-point calculations to estimate the excited state S_1_ at the S_0_ optimized geometry, and (iii) geometry optimization of the first excited state (S_1_) in both solvents. All calculations were performed at the CAM-B3LYP/def2-TZVP level with implicit solvation where applicable. At ground state, the dipole moment for **1** increased when going from THF to EtOH (Δμ = +0.536 D) (see Table 3). This behavior reflects stabilization of a more polarized electronic distribution by the solvent environment. The moderate increase between THF (ε ≈ 7.6) and EtOH (ε ≈ 24.5) is consistent with their difference in dielectric constant, with EtOH providing stronger stabilization. The dipole moment analysis for the excited state of compound **1** was performed in two different conditions/geometries in THF and EtOH. First, dipole moments for **1** were obtained from vertical excitation TD-DFT single point calculations on the S_0_ optimized geometry. Dipole moments were also computed from the optimized geometry of the first excited state (S_1_) for **1**. A comparative analysis of dipole moments differences between the ground and the excited states for **1** can be found in Table 3. As the medium’s polarity increases, moving from THF to EtOH, the dipole moment difference with respect to the ground state increases. The solvent helps to stabilize the electron density at the excited state, making the molecules more polar than the ground state. The increase of ∼9–10 D in the excited state strongly indicates an intramolecular charge-transfer (ICT) state that is heavily stabilized by dielectric polarization. These computational results are consistent with solvatofluorochromism behavior found by experimental evidence. Finally, geometry relaxation slightly enhances polarity but does not drastically alter the excited-state nature. In solvents, structural relaxation produces only marginal changes in dipole magnitude as Δμ for S_1_(vertical) → S_1_(optimized): + 0.011 D in THF and Δμ for S_1_(vertical) → S_1_(optimized): + 0.238 D in EtOH. Thus, the electronic polarization dominates over structural reorganization to explain the difference in dipole moment between the excited and the ground states.^52^

**Table 3:** Computed Dipole Moments (in Debye) for **1** in Two Different Environments at Ground and Excited States

State	THF	EtOH
S_0_ optimized geometry	9.210	9.746
S_1_ at S_0_ optimized geometry^a^	18.487	19.466
S_1_ optimized geometry	18.498	19.704

a The vertical excitation TD-DFT single-point calculation was performed.

#### 2.1.7 Electrochemical Studies

The cyclic voltammograms of compounds **1**–**4**, as well as their precursor (apocynin), were recorded in order to evaluate their behavior in reduction and oxidation processes (Figure 8). The electrochemical profiles of compounds **1**–**4** were compared with that of apocynin, which exhibits an oxidation peak at 1.459 V, associated with the irreversible oxidation of the hydroxyl group.^53^ Compounds **1**–**4** exhibit voltametric profiles different from those of apocynin, indicating that the incorporation of the conjugated α,β-unsaturated backbone and different donor groups significantly alters the electronic properties and redox accessibility of the system. In general, all derivatives show lower oxidation potential than apocynin, suggesting stabilization of the phenolic group after its integration into a more extended π-conjugated structure. This stabilization is consistent with the theoretical results, where electronic delocalization along the D−π–A–D system reduces charge localization on the phenolic oxygen.

**Figure 8:**
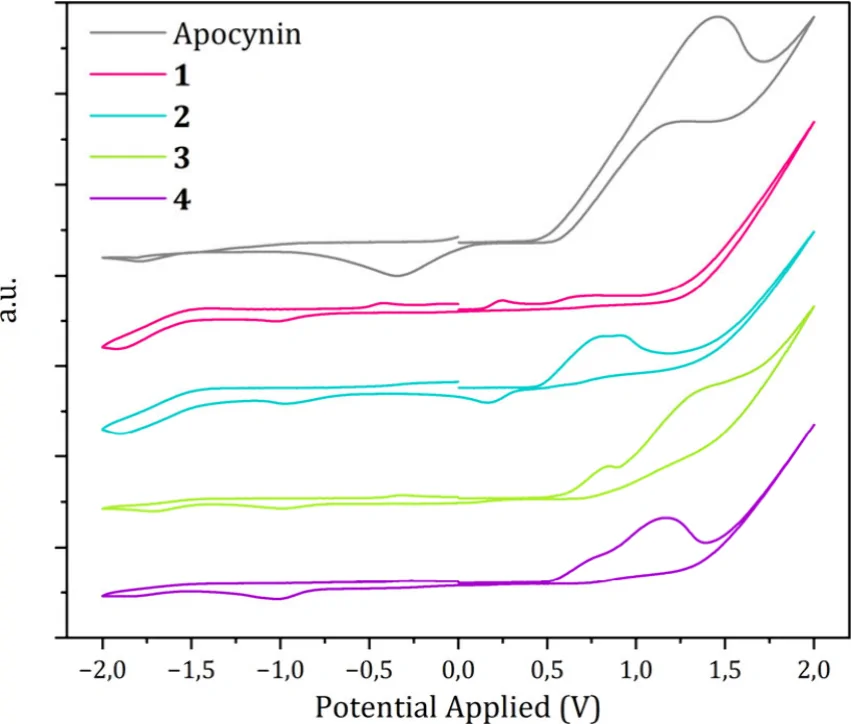
Cyclic voltammograms of compounds **1-4** and apocynin in THF solution containing 0.1 M TBAPF_6_ measured at a scan rate of 100 mV s^–1^.

As shown in Table 4, compound **1** presents two oxidation processes at 0.246 and 0.692 V. The latter shifted toward less positive values compared to apocynin, indicating an increase in the π-system electron density, attributable to an extended conjugation which makes the system easier to oxidize, probably due to a further stabilization of charges of the oxidized species in **1**. The lower potential observed at 0.246 V could be assigned to the oxidation of the other electron-donor unit, the phenyldimethylamino group.^54^ For compound **2**, two oxidation peaks can be discerned as well, the first oxidation peak at 0.805 V assigned to the oxidation of triphenylamine unit which shows an anodic shifting in comparison to the first oxidation peak of compound **1**; this can be explained by the propeller-like, twisted nonplanar conformation of the triphenylamine unit, which causes the lone pair to be deeply buried and delocalized in the π-system relative to a highly exposed lone pair in aliphatic amines such as in compound **1**.^55,56^ A more positive peak at 0.910 V was also observed and is attributable to the oxidation of the apocynin unit. Similar trends can be discerned in compounds **3** and **4** exhibiting two oxidation potentials, the more positive value assigned to the oxidation potential of the apocynin unit at the OH group, and the less positive value attributed to the other electron-donor unit, that is, tetraphenylethylene and pyrene units, respectively (Figure 8). In the reduction processes of all compounds, irreversible reduction peaks were observed and can be attributed to the apocynin unit, as has been observed in related systems.^12,57^ Compound **1** exhibited an electrochemical gap value of 1.272 eV, evidencing a strong energetic proximity between the frontier orbitals, which suggests a highly polarizable system with high facility for charge-transfer processes. In the case of compound **3**, an electrochemical gap of 1.833 eV is observed, reflecting a significant stabilization of the LUMO relative to compounds **1** and **4**, in agreement with its pronounced intramolecular charge transfer (ICT) character and with the larger red shifts and Stokes shifts observed experimentally. On the other hand, compound **4** shows an electrochemical gap of 1.773 eV, suggesting a more electronically rigid system with lower ease of electronic reorganization when compared to compounds **2** and **3**.^58^

**Table 4:** Electrochemical Parameters Acquired from Voltammograms for Compounds **1**–**4** ^a^

Compound	*E*_ox,1_(V)	*E*_ox,2_(V)	*E*_red_(V)	*E*_HOMO_(eV)	*E*_LUMO_(eV)	*E*_g_ (eV)	*E*_0–0_(eV)
**1**	0.246	0.692	–1.026	–4.686	–3.414	1.272	2.802
**2**	0.805	0.918	–0.974	–5.245	–3.466	1.779	2.756
**3**	0.841	1.316	–0.992	–5.281	–3.448	1.833	2.999
**4**	0.769	1.149	–1.004	–5.209	–3.436	1.773	2.841

a *E*_HOMO_ is calculated by *E*_HOMO_ = −4.44 eV – *E*_ox 1/2_, *E*_LUMO_ was estimated by *E*_LUMO_ = −4.44 eV – *E*_red_. *E*_0–0_ was determined at the intersection of normalized absorption and emission spectra.

#### 2.1.8 Cell Viability

Motivated by the notable fluorescence properties of compounds **1**–**4**, we investigated their practical utility in live cell bioimaging and their efficient internalization in a cell line. In vitro experiments were performed using cells derived from human laryngeal epidermoid carcinoma (HEp-2), exposed to compounds **1**–**4** at concentrations of 200, 500, and 1000 μM. Stock solutions of the compounds were prepared in dimethyl sulfoxide (DMSO) and subsequently diluted in the cell culture medium, resulting in final DMSO concentrations of 1%, 2.5%, and 5% v/v during a 30 min exposure period. The fluorescence emission of compounds **1**–**4** was evaluated following excitation at 405 nm, with emission detected in the green channel (450–490 nm), allowing the fluorescence associated with the compounds to be visualized in relation to the cells (Figures 9 and 10). DAPI was used to stain the cell nuclei and identify the position of the cells. Vehicle-treated negative controls were independently evaluated for each corresponding DMSO concentration (1%, 2.5%, and 5% v/v in culture medium for 30 min) to rule out solvent-related effects. No evident alterations in cellular morphology were observed in the vehicle-treated controls, with the maximum DMSO concentration (5% v/v) being representative of the control imaging shown in Figure 9. The results indicated favorable cell staining with compound **4** at the different concentrations evaluated. Compounds **1** and **2** also exhibited good cell-associated fluorescence at low and moderate concentrations (200 and 500 μM, respectively). However, at the highest concentration (1000 μM), compound **1** displayed rod-like aggregates that spatially overlapped with the cells, whereas compound **2** showed a more diffuse distribution throughout the cell culture medium (Figure 9). Compound **3** showed limited cell-associated fluorescence at all tested concentrations, which could be attributed to the highly hydrophobic nature of its tetraphenylethylene fragment compared with the other electroactive units present in compounds **1**, **2**, and **4**.^59^ Figure 10 shows brightfield images of the cell culture media studied along with merged images of DAPI and compounds **1**–**4** (at 500 μM), providing greater clarity of the fluorescence and cell-staining patterns observed in HEp-2 cells.

**Figure 9:**
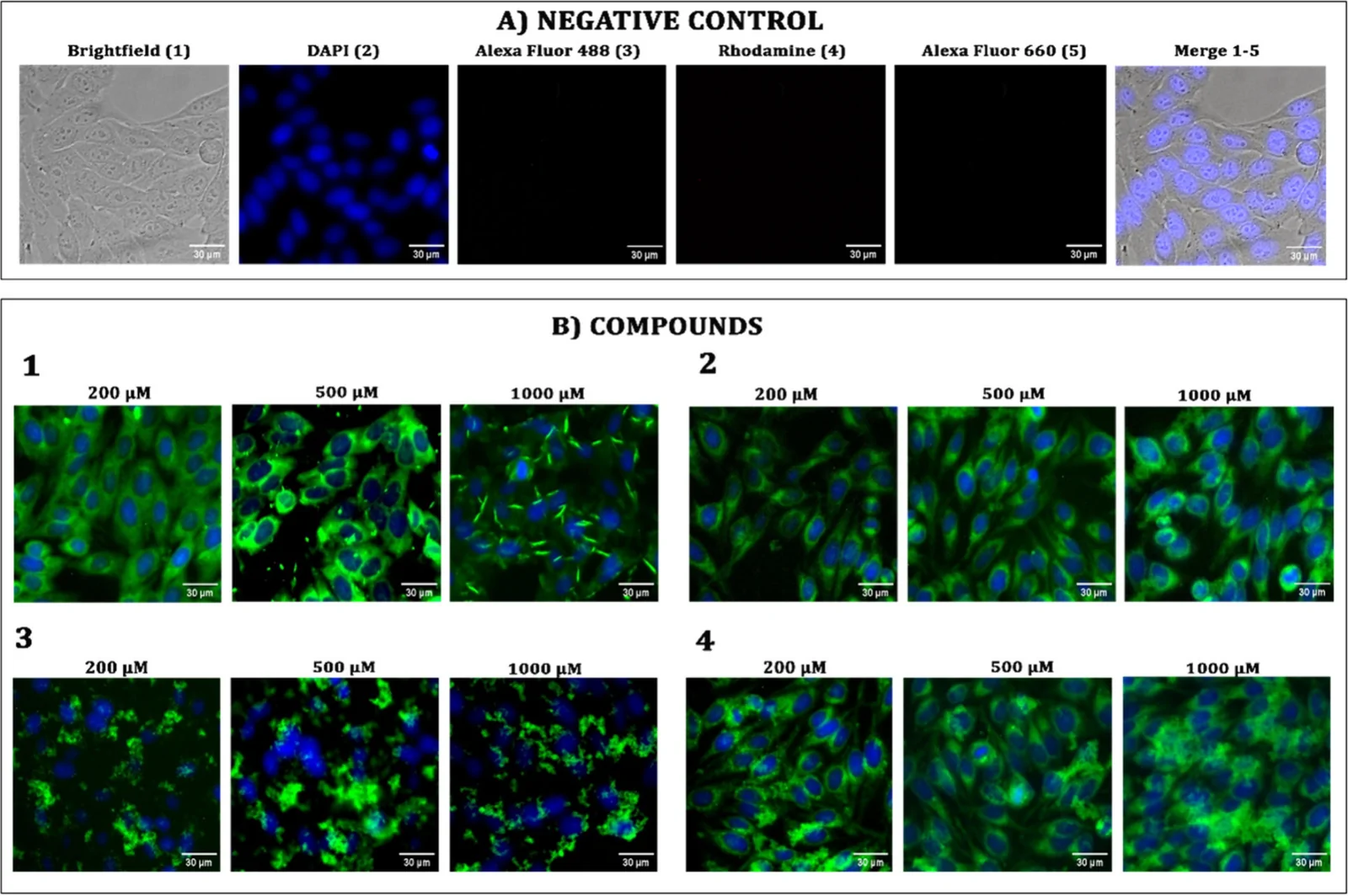
Confocal fluorescence microscopy images of HEp-2 cells (excited at 405 nm, collecting green channel emission at 450–490 nm). (A) Negative control (cells treated with vehicle DMSO 5% v/v for 30 min): Brightfield (1), DAPI nuclear stain (2), Alexa Fluor 488/green channel (3), Rhodamine channel (4), Alexa Fluor 660 channel (5), and merge (1–5). (B) Merged images of DAPI (blue, nuclei) and compound emission (green channel) for cells incubated with compounds **1**–**4** at 200, 500, and 1000 μM. Scale bars: 30 μm.

**Figure 10:**
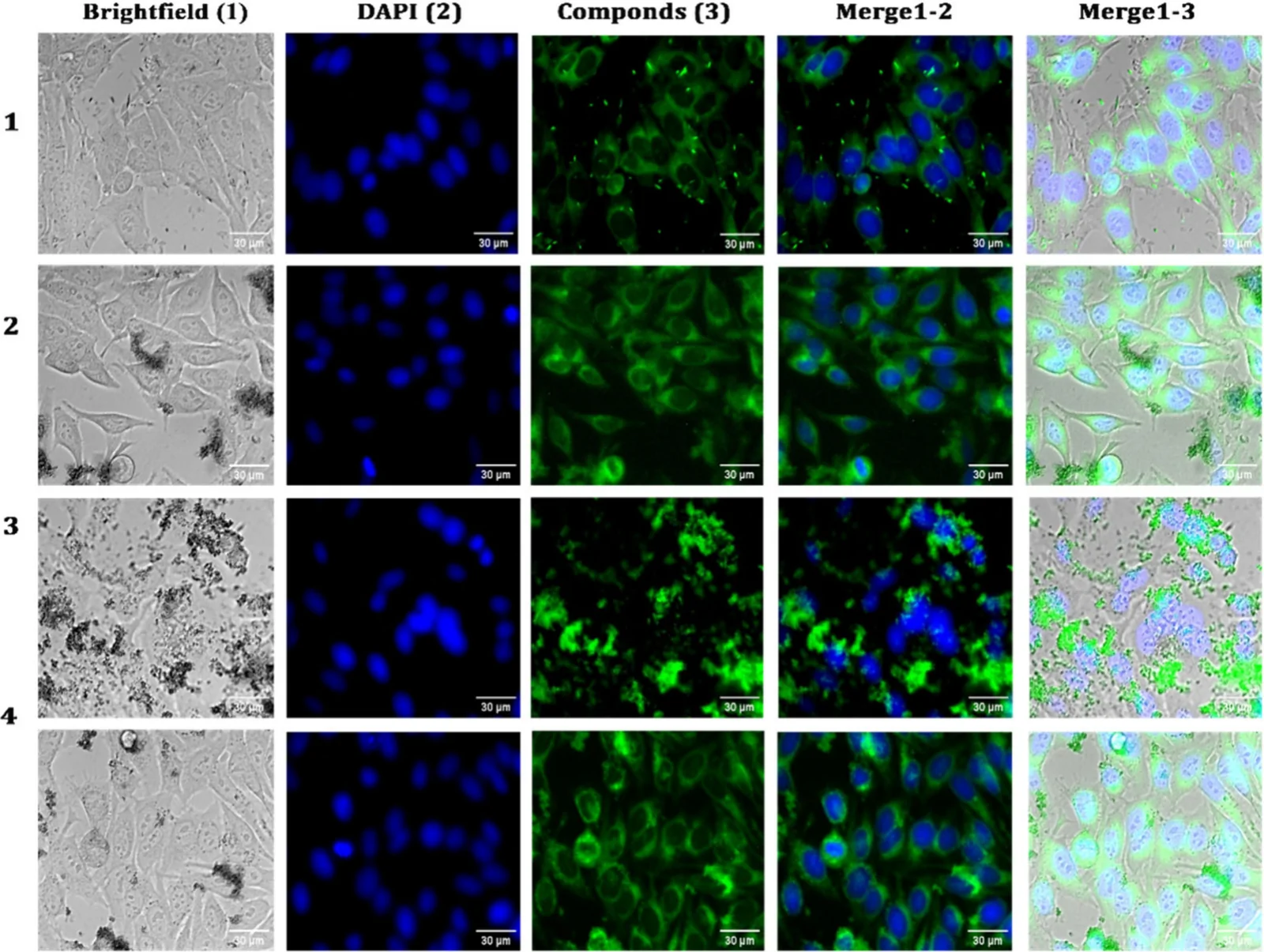
Confocal fluorescence microscopy images of HEp-2 cells loaded with compounds **1**–**4** (500 μM) excited at 405 nm and collecting green channels (450–490 nm). Scale bars: 30 μm.

Spectral cytometer analysis of morphological parameters (FSC and SSC) (Figure S15) indicated that the cells maintained integrity and homogeneity in size and complexity, ruling out significant morphological alterations due to incubation with the compounds. This suggests that the observed differences in fluorescence are the result of specific interactions between the compounds and the cells rather than adverse cellular changes. All compounds (**1**–**4**) showed a significant increase in fluorescence intensity compared with the control (Figure S16), demonstrating absorption or specific binding to the cells. The negative control showed only basal autofluorescence with no remarkable signals in the analyzed channels, confirming that the signals detected for the compounds are specific and not artifacts. The observed spectral variability indicates that each compound has a unique fluorescence profile, enabling its potential differential use in biomolecular applications depending on the predominant spectral channel.

Furthermore, the MTT assay in HEp-2 cells (24 h) indicated that all four compounds exhibit a pattern consistent with concentration-dependent toxicity: at higher doses, the apparent metabolic activity/viability decreases, and as the concentration is reduced, the response progressively recovers and approaches the negative control. This behavior supports a dose-dependent effect within the tested range (Table 5).

**Table 5:** Cell Viability (%) of Compounds **1**–**4** at Different Concentrations (μM)

μM treatment	**1**	**2**	**3**	**4**
Vancomycin (1 μM)	37.3 ± 1.3	50.7 ± 18.2	40.2 ± 9.1	29.8 ± 3.3
100	70.0 ± 5.0	57.3 ± 23.0	51.5 ± 2.5	73.8 ± 3.4
50	74.9 ± 3.7	48.2 ± 11.7	55.5 ± 3.5	80.0 ± 7.5
25	83.4 ± 3.5	76.7 ± 7.6	69.8 ± 6.5	89.6 ± 4.2
12.5	85.0 ± 1.1	96.7 ± 3.1	75.8 ± 8.2	92.4 ± 9.1
6.25	91.1 ± 5.4	98.0 ± 10.1	77.8 ± 14.5	92.3 ± 4.3
3.125	98.6 ± 6.5	105.9 ± 14.9	93.8 ± 8.6	92.8 ± 8.3
Control -	98.7 ± 4.6	100.7 ± 7.8	100.3 ± 7.4	104.9 ± 10.0

The IC_50_ values confirmed clear differences in cytotoxic potency among compounds **1**–**4**. Compound **2** was the most cytotoxic in HEp-2 cells, with an IC_50_ = 48.27 μM, suggesting that moderate concentrations are sufficient to reduce the MTT signal by 50%. Compound **3** showed a lower cytotoxic potency compared to compound **2**, with an IC_50_ = 93.77 μM, indicating that higher concentrations are required to achieve the same effect. In contrast, compounds **1** and **4** did not reach a 50% reduction within the evaluated range (IC_50_ > 100 μM), suggesting a more favorable tolerance profile in HEp-2 cells under these experimental conditions.

Although the IC_50_ results indicate that compounds **1**–**4** generally do not exhibit marked cytotoxic potency against HEp-2 tumor cells, this behavior does not rule out their potential biological utility. On the contrary, the combination of low toxicity, cellular uptake capacity, and fluorescent emission suggests that these compounds could be explored as core structures for the development of cellular markers or diagnostic tools. In this context, their limited impact on cell viability could represent an advantage, as it may enable their potential use in labeling, visualization, or cell-tracking applications without significantly compromising the integrity of the evaluated cells.

## 3 Conclusions

Four electroactive chalcones derived from apocynin (**1**–**4**) were successfully synthesized and characterized through a Claisen–Schmidt condensation, featuring D−π–A–D type push–pull molecular arrangements, in high yields. Photophysical studies in various solvents revealed large Stokes shifts (3,000–9,000 cm^–1^) and a pronounced positive solvatofluorochromic behavior, indicating highly polar excited states. Fluorescence studies performed on THF/water mixtures showed that nitrogen-based compounds **1** and **2** exhibited a marked ACQ effect, while compounds **3** and **4** showed a notable AIE behavior. Viscosity studies showed that all compounds **1**–**4** enhanced their emission intensity as the viscosity increases, particularly on compounds **2** and **3**, where the restriction of intramolecular motions is addressed by the presence of all phenyl rotors in the viscous medium. Cellular bioimaging experiments using compounds **1**–**4** revealed that all of them served as good fluorescent probes for cell staining, except for compound **3**. DFT/TD-DFT theoretical calculations and cyclic voltammetry experiments evidenced a good electronic communication for all compounds **1**–**4**. All these findings demonstrate that the apocynin-based α,β-unsaturated systems constitute versatile luminescent platforms with tunable photophysical properties, suitable for applications in cellular bioimaging and the development of efficient fluorescent materials.

## 4 Experimental Section

### 4.1 Chemistry

#### 4.1.1 General

Nuclear magnetic resonance (NMR) spectra were recorded on a Bruker Avance II 400 spectrometer operating at 400 MHz for ^1^H and 101 MHz for ^13^C, using deuterated DMSO (DMSO-*d*_*6*_) as solvent. Chemical shifts (δ) are reported in parts per million (ppm) relative to residual solvent signals (DMSO-*d*_*6*_: δ_H_ 2.50, δ_C_ 39.52). Electron impact (EI) mass spectra were acquired on a Shimadzu GCMS-QP2010 Ultra gas chromatograph–mass spectrometer at 70 eV. Thermogravimetric analysis (TGA) was performed using a LanzettaRengifo TGA50 instrument (model Q50-1946) under a constant nitrogen atmosphere. UV–Vis absorption spectra were recorded on a JASCO V-730 spectrophotometer, and fluorescence spectra were obtained using a JASCO FP-8500 spectrofluorometer.

#### 4.1.2 General Procedure for the Synthesis of Chalcones Derived from Apocynin **1**–**4**

The respective aryl aldehyde (200 mg) and apocynin (1 equiv) were dissolved in 5 mL of methanol. Then, 0.5 mL of an aqueous solution of KOH (3 pellets) was added, and the mixture was stirred at room temperature for 48 h. Then, 10 mL of water was added, and the mixture was neutralized with acetic acid. The precipitate that formed was filtered and washed with water and cold methanol. The desired compound was purified by silica gel column chromatography using dichloromethane as an eluent.

##### 4.1.2.1 3-(4-(Dimethylamino)­phenyl)-1-(4-hydroxy-3-methoxyphenyl)­prop-2-en-1-one (**1**)

Compound **1** was obtained as an orange solid (325 mg, yield: 82%). FTIR (ATR) ν = [3201] (OH), [1635] (C=O) cm^–1^. ^1^H NMR (400 MHz, DMSO-*d*_6_) δ 9.69 (s, 1H), 7.73 (dd, *J* = 8.3, 1.6 Hz, 1H), 7.69 (d, *J* = 8.8 Hz, 2H), 7.65–7.58 (m, 3H), 6.89 (d, *J* = 8.3 Hz, 1H), 6.74 (d, *J* = 8.8 Hz, 2H), 3.87 (s, 3H), 3.00 (s, 6H) ppm. ^13^C NMR (101 MHz, DMSO-*d*_6_) δ 187.2, 152.5, 152.2, 148.3, 144.1, 130.9, 130.2, 123.7, 122.8, 116.7, 115.5, 112.2, 112.0, 56.1, 39.4 ppm. MS-EI (70 eV) *m*/*z* (%): 297 (100), 174 (27), 146 (23), 121 (39), 83 (20), 57 (33), 43 (31).

##### 4.1.2.2 3-(4-(Diphenylamino)­phenyl)-1-(4-hydroxy-3-methoxyphenyl)­prop-2-en-1-one (**2**)

Compound **2** was obtained as a yellow solid (241 mg, yield: 78%). FTIR (ATR) ν = [3537] (OH), [1647] (C=O) cm^–1^. ^1^H NMR (400 MHz, DMSO-*d*_6_) δ 10.01 (s, 1H), 7.78–7.71 (m, 4H), 7.67–7.60 (m, 2H), 7.40–7.33 (m, 4H), 7.17–7.08 (m, 6H), 6.95–6.88 (m, 3H), 3.87 (s, 3H) ppm. ^13^C NMR (101 MHz, DMSO-*d*_6_) δ 187.4, 152.2, 149.8, 148.2, 146.8, 142.9, 130.6, 130.2, 128.5, 125.6, 124.7, 123.9, 121.4, 120.0, 115.5, 112.1, 56.1 ppm. MS-EI (70 eV) *m*/*z* (%): 421 (100), 267 (8), 245 (10), 196 (9), 167 (14), 151 (9).

##### 4.1.2.3 1-(4-Hydroxy-3-methoxyphenyl)-3-(4-(1,2,2-triphenylvinyl)­phenyl)­prop-2-en-1-one (**3**)

Compound **3** was obtained as a yellow solid (244 mg, yield: 87%). FTIR (ATR) ν = [3496] (OH), [1649] (C=O) cm^–1^. ^1^H NMR (400 MHz, DMSO-*d*_6_) δ 10.05 (s, 1H), 7.83 (d, *J* = 15.1 Hz, 1H), 7.75 (dd, *J* = 8.4, 2.0 Hz, 1H), 7.64 (d, *J* = 8.3 Hz, 2H), 7.61 (s, 1H), 7.59 (d, *J* = 15.1 Hz, 1H), 7.18–7.10 (m, 9H), 7.04–6.96 (m, 8H), 6.90 (d, *J* = 8.4 Hz, 1H), 3.86 (s, 3H) ppm. ^13^C NMR (101 MHz, DMSO-*d*_6_) δ 187.4, 152.4, 148.2, 145.8, 143.8, 143.5, 143.3, 142.6, 141.9, 141.2, 140.5, 133.5, 131.7, 131.1, 130.9, 129.9, 128.8, 128.4, 128.3, 127.3, 127.2, 127.0, 126.3, 124.2, 122.4, 115.5, 112.1, 56.2 ppm. MS-EI (70 eV) *m*/*z* (%): 508 (100), 253 (38), 151 (28), 123 (11).

##### 4.1.2.4 1-(4-Hydroxy-3-methoxyphenyl)-3-(pyren-1-yl)­prop-2-en-1-one (**4**)

Compound **4** was obtained as a yellow solid (300 mg, yield: 91%) FTIR (ATR) ν = [3479] (OH), [1643] (C=O) cm^–1^. ^1^H NMR (400 MHz, DMSO-*d*_6_) δ 8.81 (d, *J* = 6.2 Hz, 1H), 8.78 (s, 1H), 8.60 (d, *J* = 9.4 Hz, 1H), 8.41–8.30 (m, 4H), 8.28–8.19 (m, 3H), 8.11 (t, *J* = 7.7 Hz, 1H), 7.89 (dd, *J* = 8.3 Hz, 1.5 Hz, 1H), 7.69 (s, 1H), 6.92 (d, *J* = 8.3 Hz, 1H), 3.89 (s, 3H) ppm. ^13^C NMR (101 MHz, DMSO-*d*_6_) δ 186.7, 156.5, 149.1, 138.0, 132.5, 131.4, 130.7, 129.8, 129.3, 129.1, 128.9, 127.9, 127.7, 127.1, 126.6, 126.3, 125.7, 125.4, 125.1, 124.6, 124.3, 122.8, 116.0, 112.1, 56.1 ppm. MS-EI (70 eV) *m*/*z* (%): 378 (66), 289 (10), 226 (65), 202 (23), 151 (100), 123 (21), 57 (26).

### 4.2 Computational Details

At first, the tridimensional structure for all compounds under study were built using RDKit.^60^ Second, a fast geometry relaxation in vacuum was performed with GFN2-xTB^61^ (through Orca 6.1.0 ^62^ in order to prepare suitable geometries for high-level calculations. Next, a more rigorous geometry optimization was carried out using the ωB97X-D3­(BJ)/def2-TZVP level of theory in vacuum with ORCA 6.1.0. The RIJCOSX approximation for Coulomb and HF Exchange in the hybrid functional was used as default in all DFT calculations. In complement, frequency calculations were performed to confirm the identification of minimum point geometries in the Potential Energy Surface (PES) of each molecule (no negative frequencies were found). Finally, single-point TD-DFT calculations, using the same level of theory and the optimized geometries from the ground state DFT calculations, were performed to collect information on the excited states for the compounds in the study. The TD-DFT experiments were run without the Tamm–Dancoff approximation (TDA) and using the linear response conductor-like polarizable continuum (LR-CPCM) model^63^ to include solvent effects on electronic transitions for toluene, THF, dichloromethane, acetone, DMF, DMSO, acetonitrile, and ethanol. In order to compute the vertical excitation energies, the nonequilibrium variant of LR-CPCM was used on the implicit-solvent TD-DFT calculations.

### 4.3 Electrochemical Characterization

Cyclic voltammetry (CV) was performed on a potentiostate/galvanostate Autolab PGSTAT302N. The electrochemical cell was configured to work with three electrodes: a platinum disk, platinum wire, and Ag/AgCl as working, counter, and reference electrodes, respectively. Prior to each voltammetry measurement the cell was degassed under an argon atmosphere. The experiment was carried out at room temperature and scan rate of 100 mVs^– 1^. The supporting electrolyte was a solution of 0.1 M tetrabutylammonium hexafluorophosphate (TBAPF_6_) in THF.

### 4.4 Cytotoxicity Assays and Fluorescence Studies

#### 4.4.1 Cytotoxicity Assays

Cytotoxicity was assessed using the MTT colorimetric assay in HEp-2 cells after 24 h of exposure. Cells were seeded in 96-well plates at a density of 1.0 × 10^4^ cells/well and allowed to adhere until an appropriate confluence was reached. Subsequently, cells were treated with the corresponding experimental conditions for 24 h. Controls included a negative control (vehicle 0.5% v/v DMSO) and a positive control (vancomycin, 1 μM). The experiment was performed in three independent assays, each with three technical replicates per condition. After the exposure period, MTT was added to each well and plates were incubated in the dark to allow formazan crystal formation. Formazan was then solubilized, and absorbance was measured using a microplate reader at 570 nm. Cell viability (%) was calculated by normalizing absorbance values to the negative control (100%) and reported as mean ± standard deviation from independent assays.

#### 4.4.2 Fluorescence Microscopy

HEp-2 cells were cultured in 24-well plates using DMEM with GlutaMAX, supplemented with 10% fetal bovine serum (FBS) and 1% penicillin–streptomycin, until reaching approximately 80% confluence. The cultures were maintained under controlled conditions at 37 °C and 5% CO_2_. Once the desired confluence was reached, the supernatant was removed and replaced with 200 μL of the treatment solutions of compounds **1**–**4**, prepared in unsupplemented DMEM with GlutaMAX. Three concentrations were tested for each compound (200 μM, 500 μM, and 1000 μM). Stock solutions were prepared in dimethyl sulfoxide (DMSO) and subsequently diluted in the culture medium, resulting in final organic solvent concentrations of 1%, 2.5%, and 5% v/v within the 200 μL treatment volume. The cells were incubated with the compounds for 30 min at 37 °C and 5% CO_2_. After the exposure time, the cells were washed twice with 1X PBS. Subsequently, the nuclei were stained with DAPI using the NucBlue Live Cell Stain ReadyProbes reagent, following the manufacturer’s recommendations. Vehicle-treated negative controls were independently evaluated using matching concentrations of DMSO (1%, 2.5%, and 5% v/v in culture medium for 30 min) to rule out solvent-related effects. Finally, the cells were imaged using an Axio Observer Z1 (ZEISS) fluorescence microscope equipped with a 40× objective, acquiring images in bright-field mode alongside specific fluorescence channels: DAPI (excitation: 335–383 nm, emission: 420–470 nm), Alexa Fluor 488 (excitation: 450–490 nm, emission: 515–4095 nm), Rhodamine (excitation: 538–562 nm, emission: 570–640 nm), and Alexa Fluor 660 (excitation: 575–625 nm, emission: 660–710 nm).

#### 4.4.3 Flow Cytometry

HEp-2 cells were cultured in a T75 flask with DMEM GlutaMAX medium, supplemented with 10% fetal bovine serum (FBS) and 1% penicillin–streptomycin, and maintained at 37 °C and 5% CO_2_ until reaching approximately 80% confluence. The medium was renewed every 48 h. Once the desired confluence was reached, the supernatant was removed and the cells were washed with 10 mL of prewarmed 1× PBS. Then, 1.5 mL of prewarmed trypsin-EDTA (Sigma) was added, and the cells were incubated at 37 °C for 2 min to detach them. After this time, trypsin was neutralized with 10 mL of DMEM GlutaMAX supplemented with 10% FBS. The resulting cell suspension was transferred to a 50 mL Falcon tube and centrifuged at 300*g* for 5 min at room temperature. The supernatant was discarded, and the cell pellet was resuspended in DMEM GlutaMAX with 10% FBS and 1% penicillin–streptomycin. Cells were counted using a Neubauer chamber, and the suspension was adjusted to 1 × 10^6^ cells/mL.

To evaluate the fluorescent properties of compounds **1**–**4**, 300 μL of the cell suspension was collected and centrifuged, and the supernatant was replaced with 300 μL of each compound at a final concentration of 200 μM prepared in unsupplemented DMEM GlutaMAX. Samples were incubated at 37 °C for 30 min in a CO_2_ atmosphere. After incubation, the cells were centrifuged again at 300*g* for 5 min, the supernatant was discarded, and the cells were washed with 1× PBS. Subsequently, the cells were resuspended in 300 μL of eBioscience Flow Cytometry Staining Buffer. A negative control consisting of unstained HEp-2 cells was included to measure autofluorescence. Samples were acquired immediately after resuspension in the staining buffer, recording approximately 20,000 events per sample. Acquisition was performed on a Cytek Northern Lights cytometer equipped with three lasers: violet (405 nm), blue (488 nm), and red (640 nm). The system is configured with a spectral detection array: 16 channels for the violet laser (V1–V16), 14 for the blue laser (B1–B14), and 8 for the red laser (R1–R8).

## Supplementary Material



## Notes

^1^H NMR, ^13^C NMR, and mass spectra of compounds **1**–**4** (Figures S1–S12); normalized absorption spectra of compounds **1**–**4** (Figure S13); Lippert–Mataga and Rettig plots of compounds **1**–**4** (Figure S14); computed E_HOMO_, E_LUMO_, and the HOMO–LUMO gap (in eV) of compounds **1**–**4** (Table S1); morphological parameters (FSC and SSC) of compounds **1**–**4** (Figure S15); flow cytometry of compounds **1**–**4** (Figure S16) (PDF)
