# Supplementary material for: Synthesis and Photophysical Properties of Electroactive Apocynin Derivatives as Potential Luminogens and Cellular Bioimaging Agents
Source: ACS Omega. 2026 Oct 26;11(44):69127–42. doi: 10.1021/acsomega.6c09229 (PMC13756723; doi:10.1021/acsomega.6c09229)
Supplement: Supplementary file 1 [file ao6c09229_si_001.pdf]

Supporting Information

Synthesis and Photophysical Properties of Electroactive Apocynin Derivatives as Potential Luminogens and Cellular Bioimaging Agents

Camila García-Aquite[a], Luis Illicachi[b], Joel José Montalvo Acosta[c], Alejandro Ortiz[a], Homero G. San-Juan-Vergara[d], Juan D. Rodríguez[e], Walter A. Martínez-De-La-Rosa[f], Daniel Insuasty[g*] and Alberto Insuasty[a*]

[a] Grupo de Investigación de Compuestos Heterocíclicos, Departamento de Química, Universidad del Valle, Cali 760032, Colombia.

[b] Grupo de Investigación en Química y Biotecnología, Facultad de Ciencias Básicas, Universidad Santiago de Cali, Cali 760035, Colombia.

[c] Facultad de Ciencias Farmacéuticas, Universidad de Cartagena, Cartagena 130015, Colombia.

[d] Departamento de Medicina, División Ciencias de la Salud, Universidad del Norte, Km 5 vía Puerto Colombia, Puerto Colombia 081007, Colombia

[e] Programa de medicina, Facultad de Ciencias de la Salud, Universidad Libre, Km 7 vía Puerto Colombia, Puerto Colombia 081007, Colombia.

[f] Grupo de Investigación en Biomedicina, Departamento de Ciencias Básicas Medicas, Universidad Metropolitana, Barranquilla 080020, Colombia.

[g] Departamento de Química y Biología, División de Ciencias Básicas, Universidad del Norte, Puerto Colombia 081007, Colombia.

**Table of contents**	**Page**
^{1}H, ^{13}C NMR spectra & mass spectra of compounds **1-4**	**2-7**
Normalized absorption spectra of compounds **1-4**	**8**
Lippert-Mataga and Rettig plots of compounds **1-4**	**8**
Computed E_{HOMO}, E_{LUMO} and the HOMO-LUMO gap (in eV) of compounds **1-4**	**8**
Morphological parameters (FSC and SSC) of compounds **1-4**	**9**
Flow cytometry of compounds **1-4**	**10**

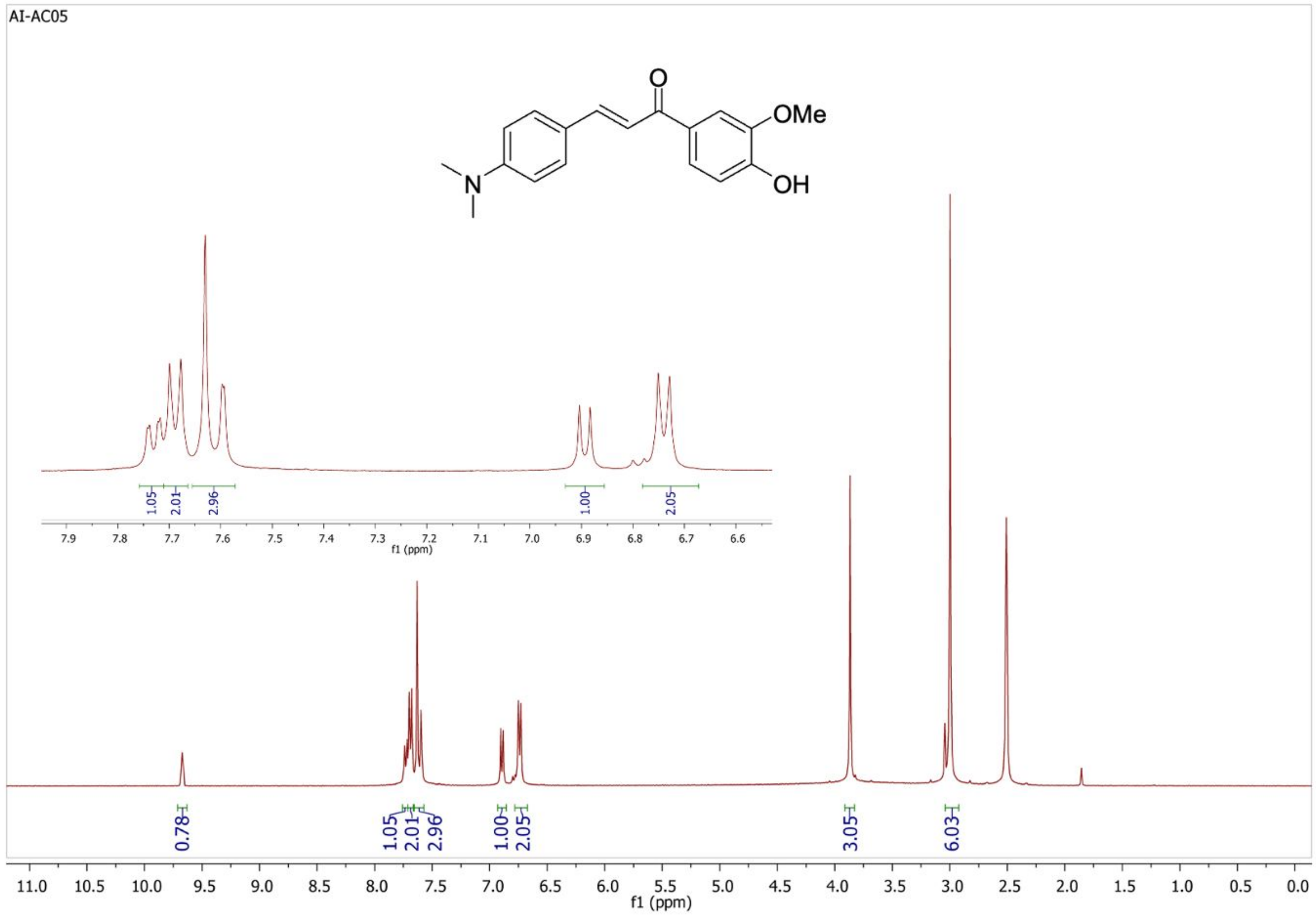

Figure S1. ^{1}H-NMR spectrum of compound **1** in DMSO-d_6 (400 MHz).

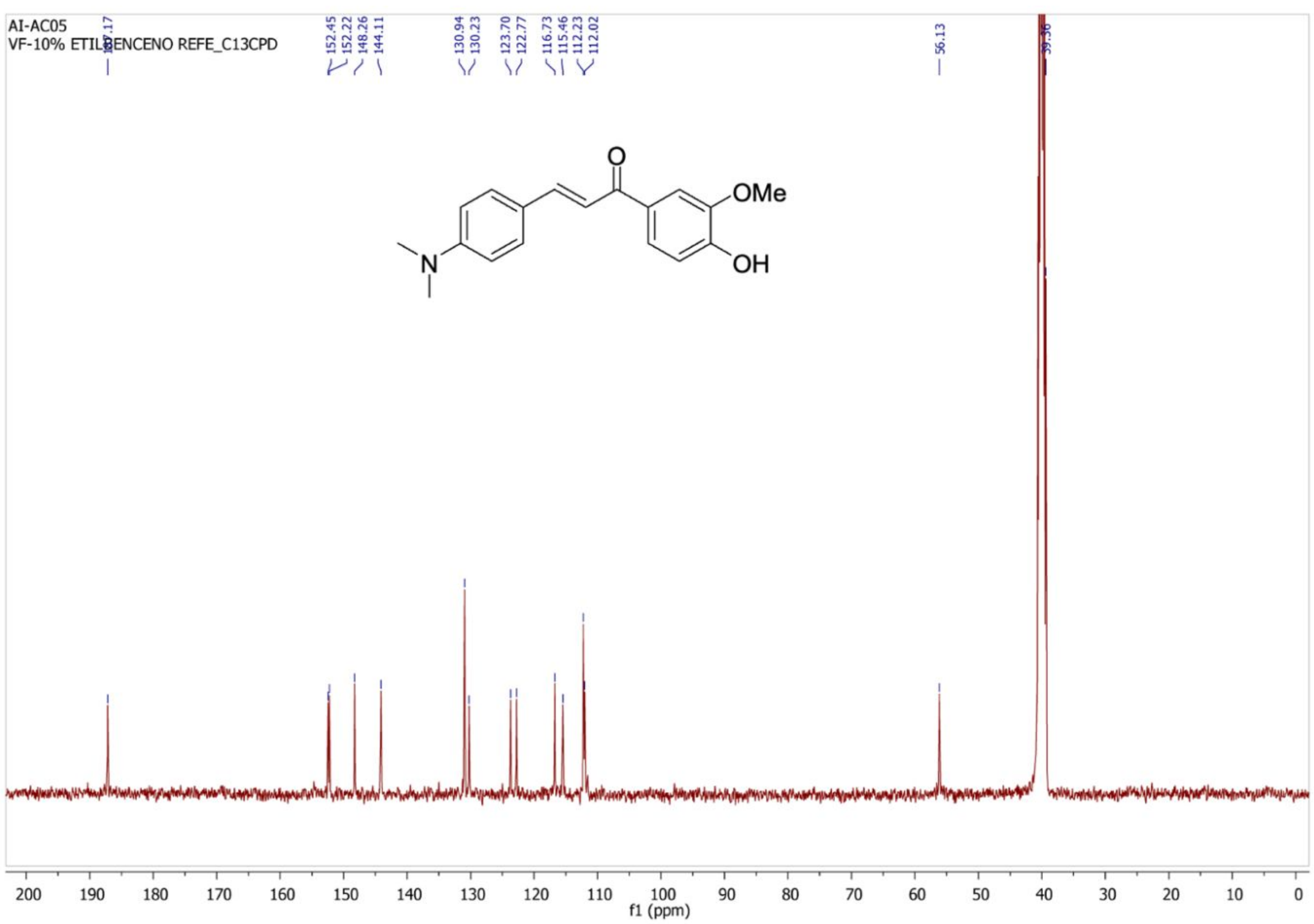

Figure S2. ^{13}C-NMR spectrum of compound **1** in DMSO-d_6 (101 MHz).

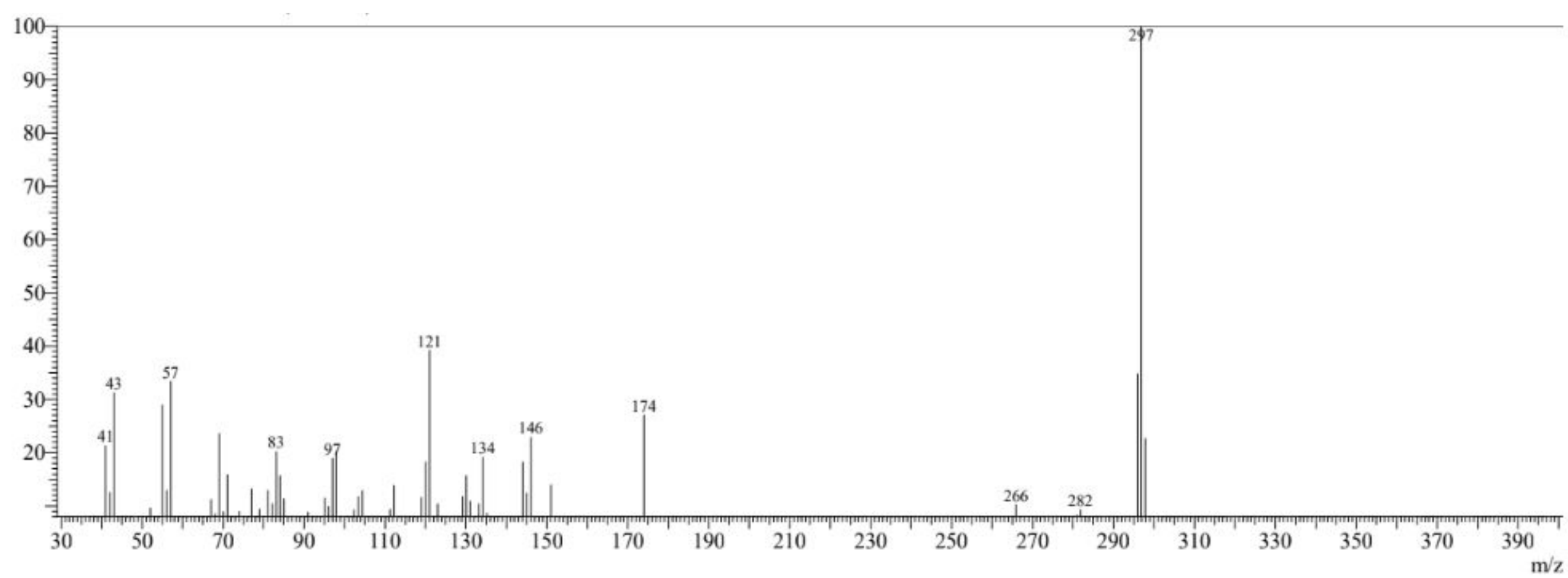

Figure S3. EI-Mass spectrum of compound **1**.

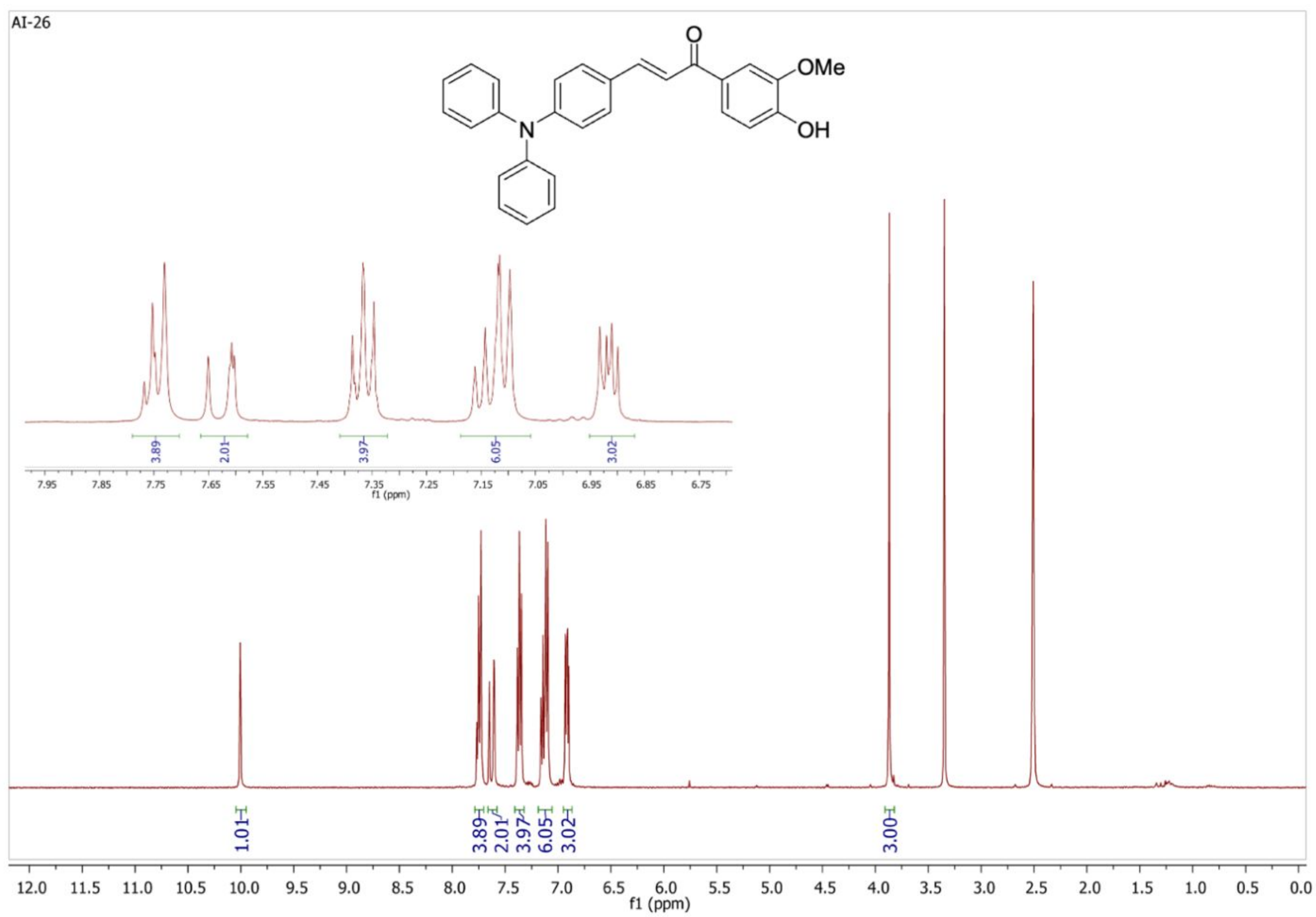

Figure S4. ^{1}H-NMR spectrum of compound **2** in DMSO-d_6 (400 MHz).

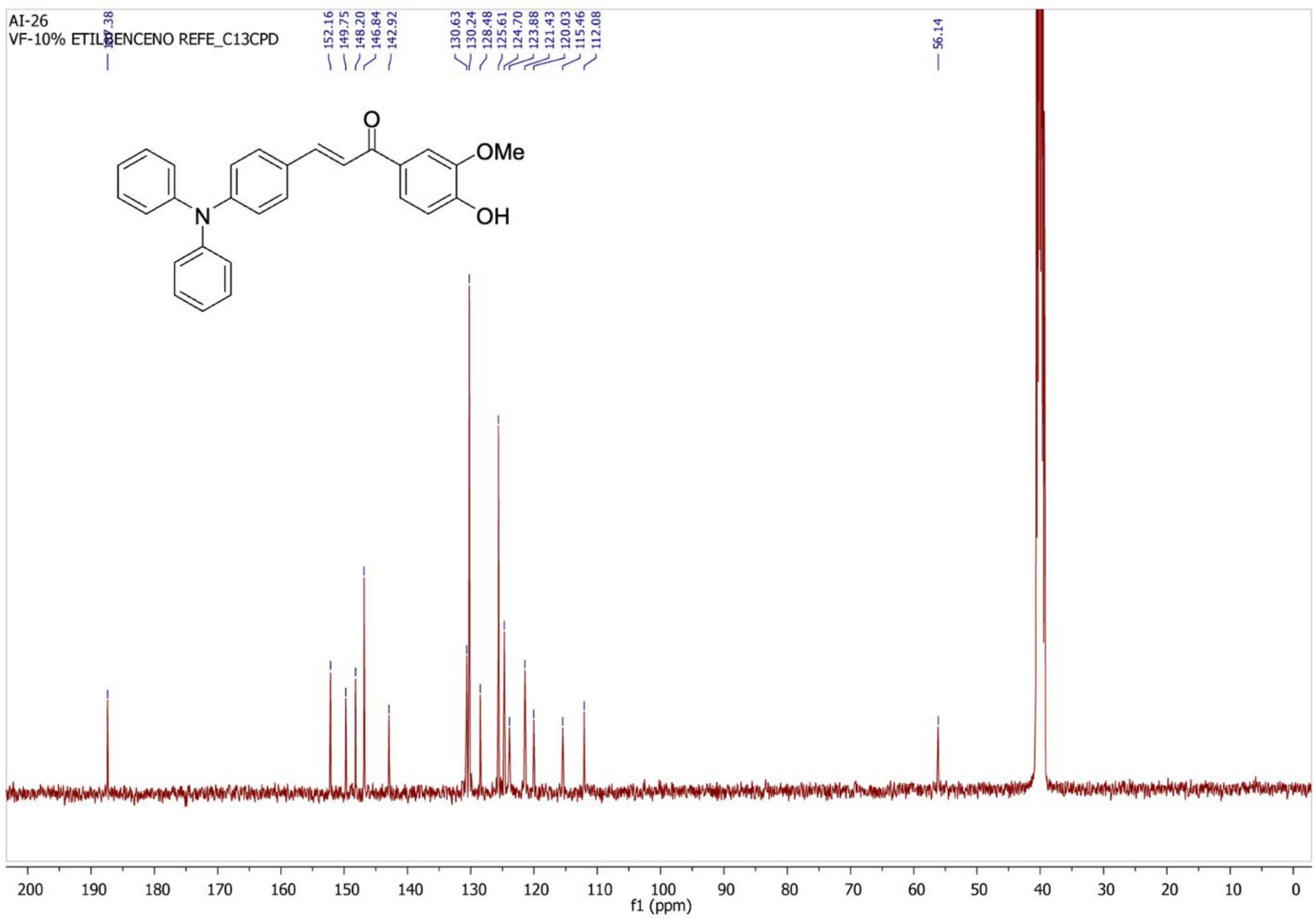

Figure S5. ^{13}C-NMR spectrum of compound **2** in DMSO-d_6 (101 MHz).

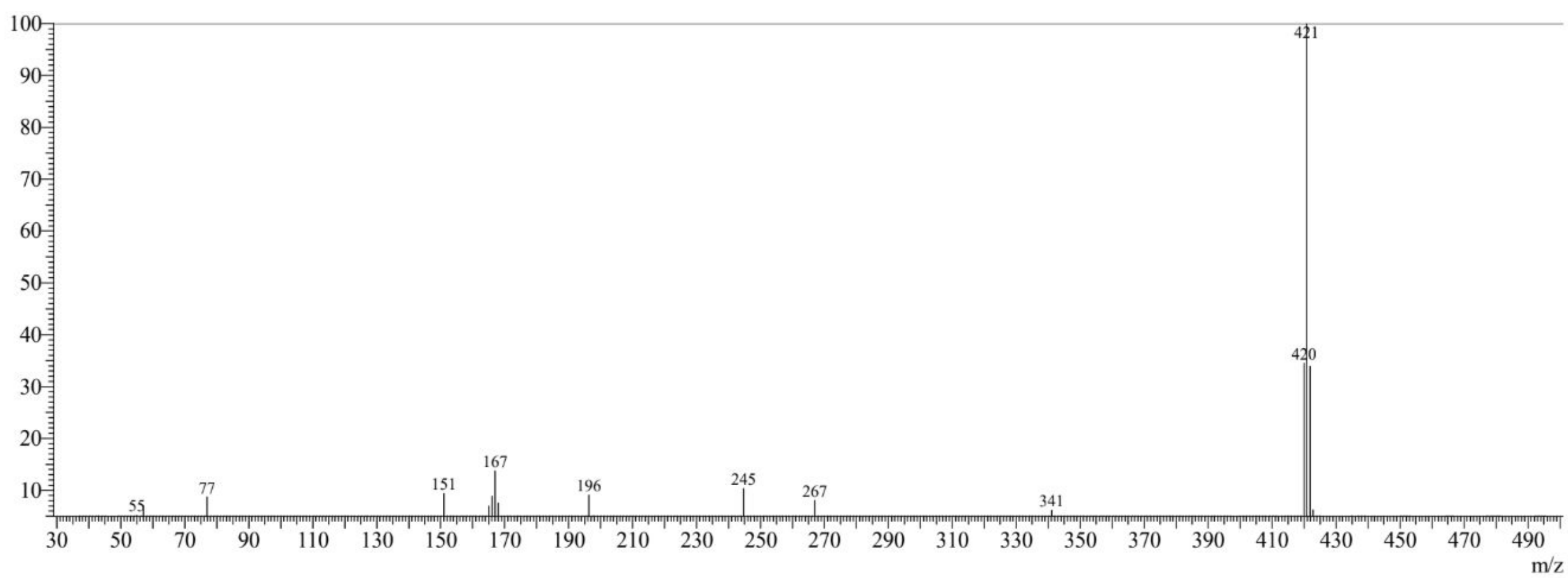

Figure S6. EI-Mass spectrum of compound **2.**

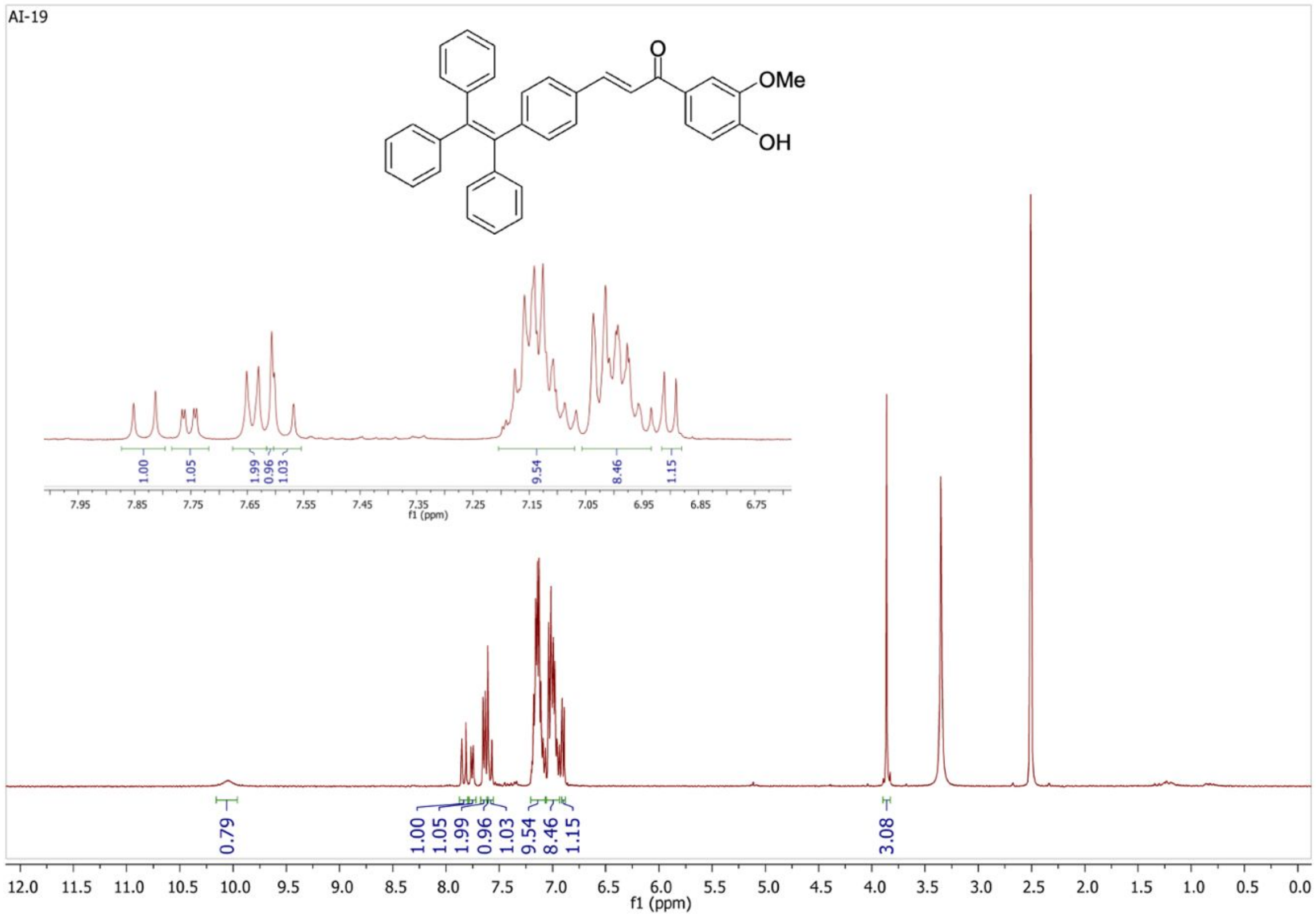

Figure S7. ^{1}H-NMR spectrum of compound **3** in DMSO-d_6 (400 MHz).

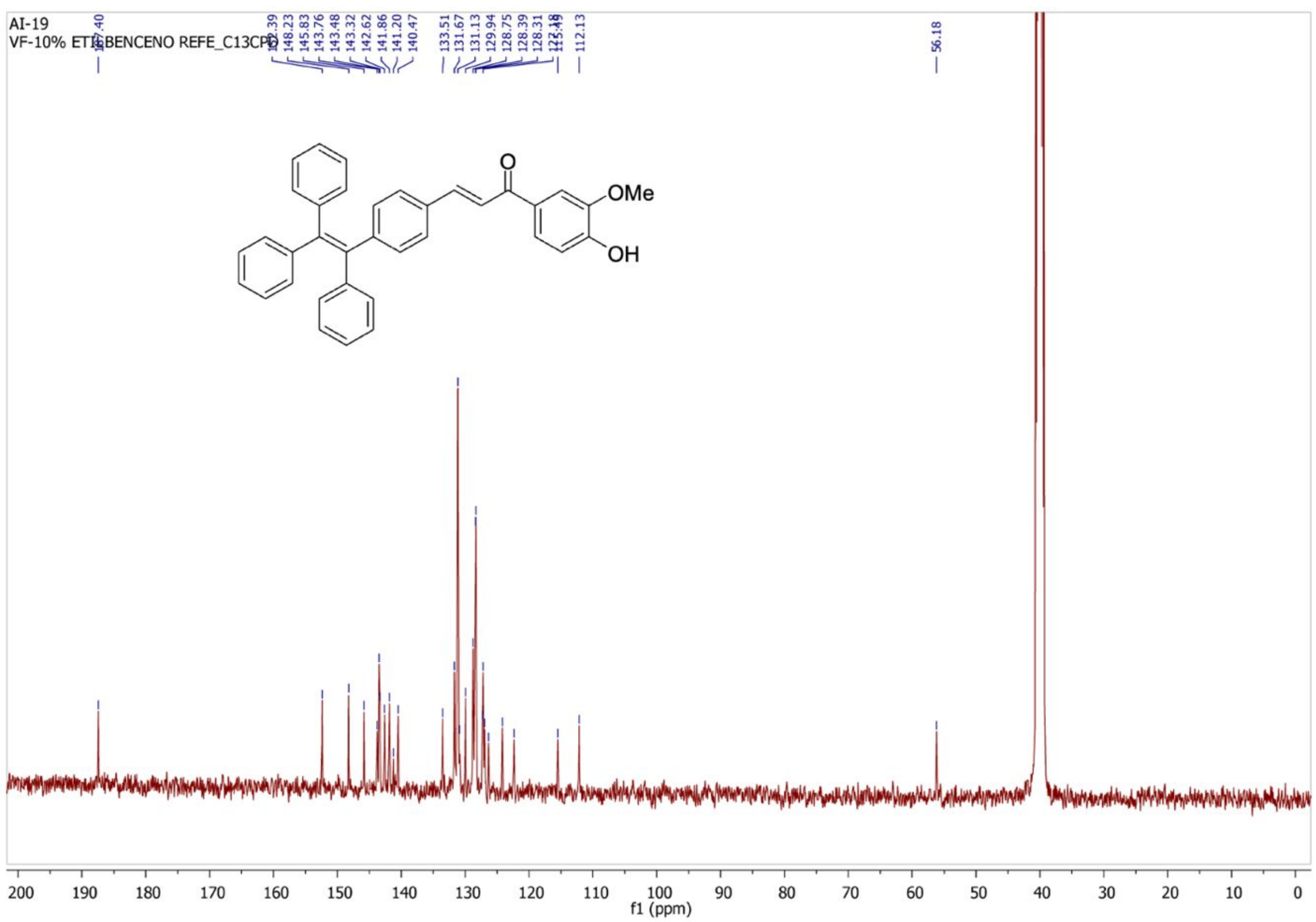

Figure S8. ^{13}C-NMR spectrum of compound **3** in DMSO-d_6 (101 MHz).

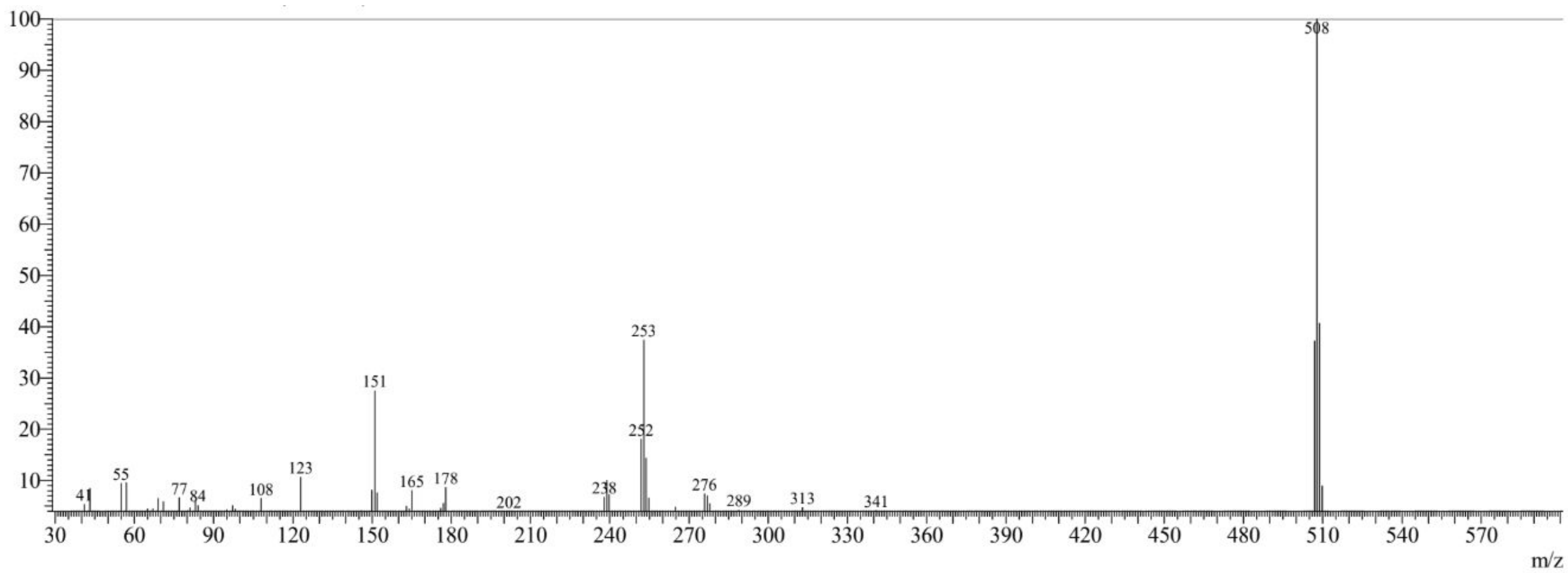

Figure S9. EI-Mass spectrum of compound **3**.

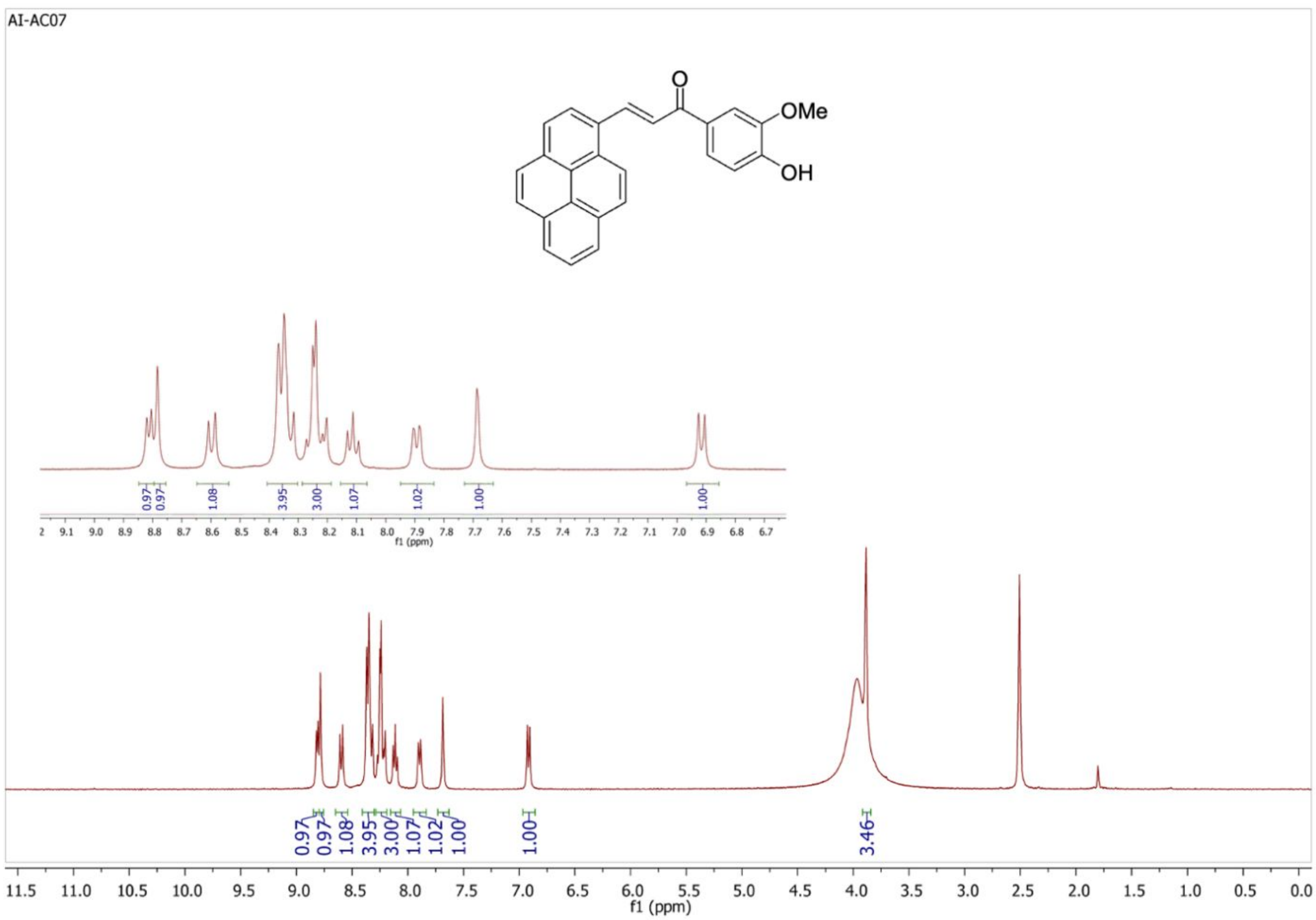

Figure S10. ^{1}H-NMR spectrum of compound **4** in DMSO-d_6 (400 MHz).

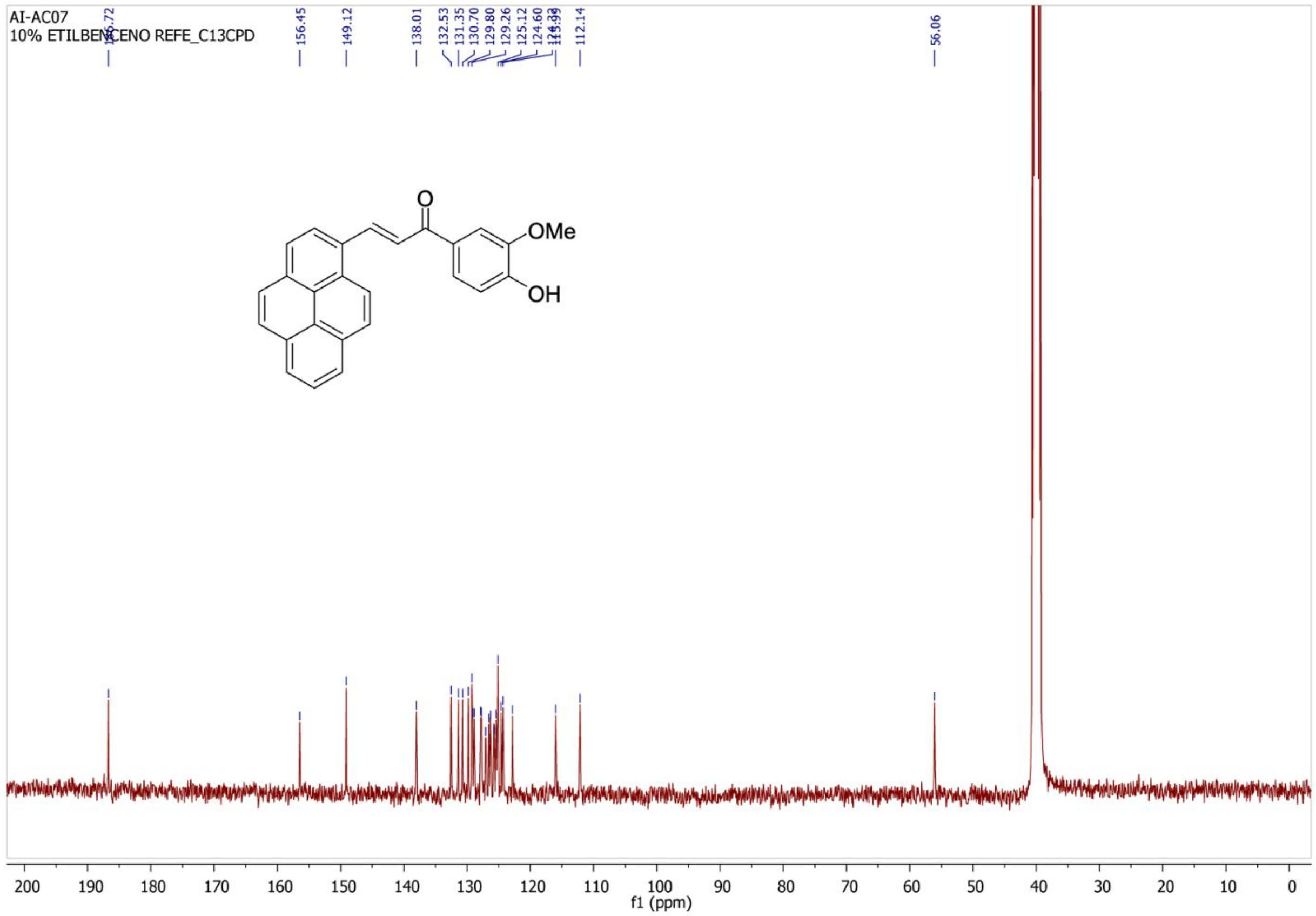

Figure S11. ^{13}C-NMR spectrum of compound **4** in DMSO-d_6 (101 MHz).

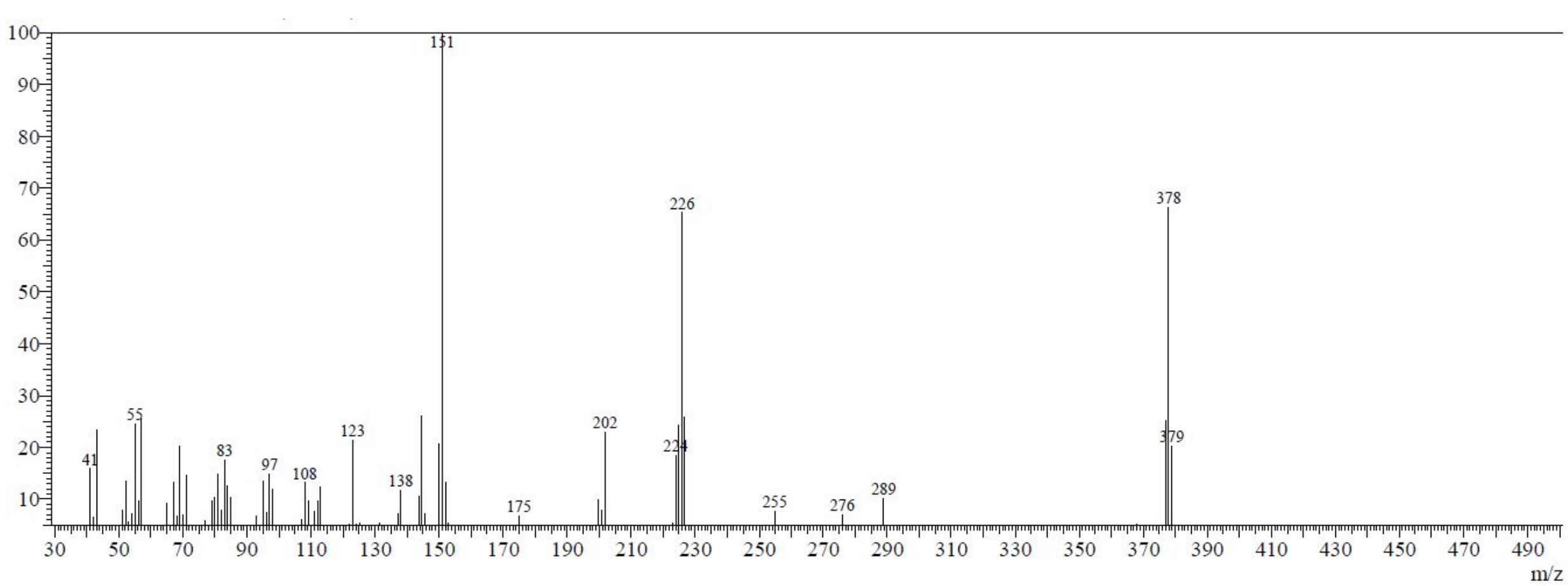

Figure S12. EI-Mass spectrum of compound **4**.

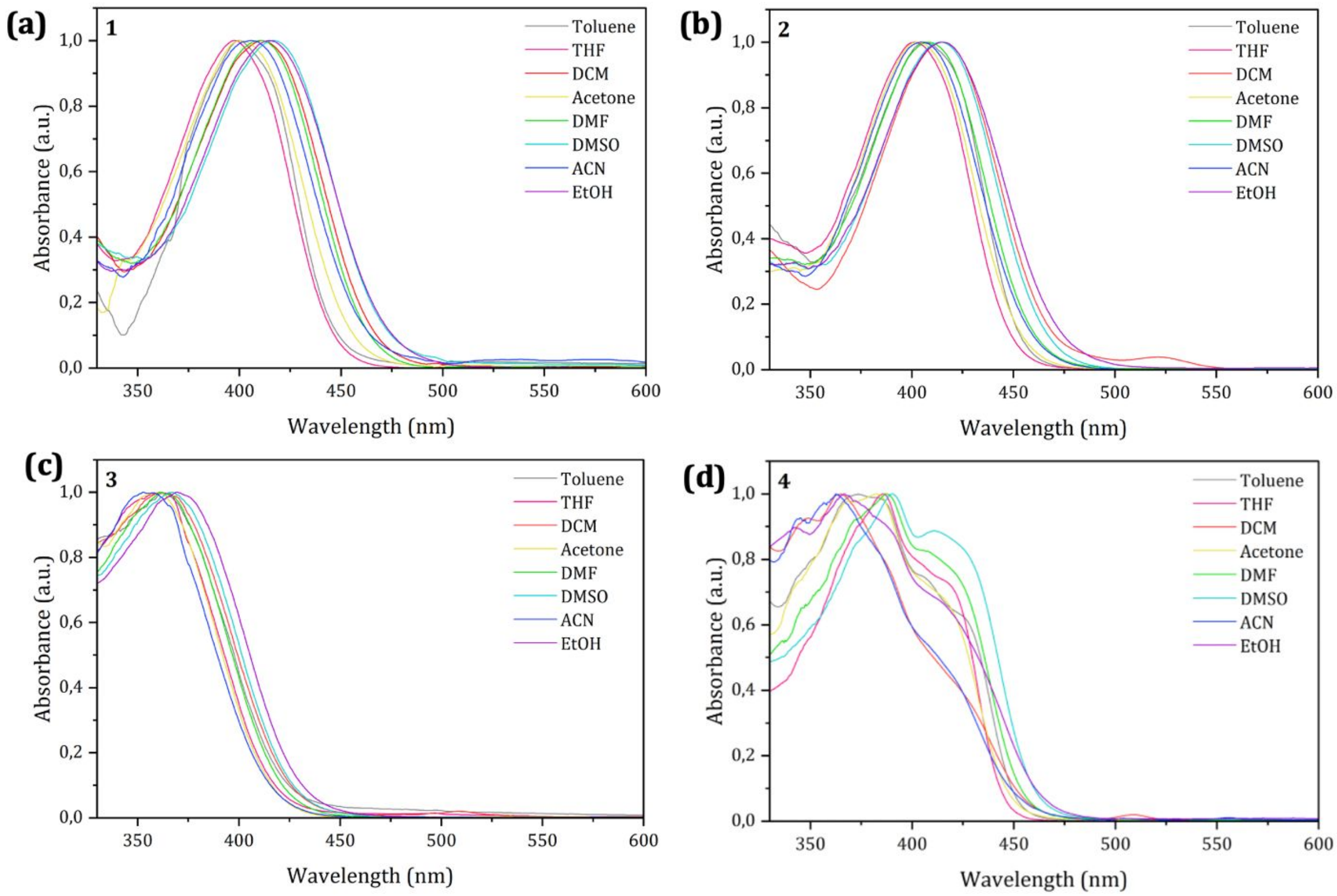

Figure S13. Normalized absorption spectra of **1** (a), **2** (b), **3** (c), and **4** (d) (5.0 μM) in different solvents.

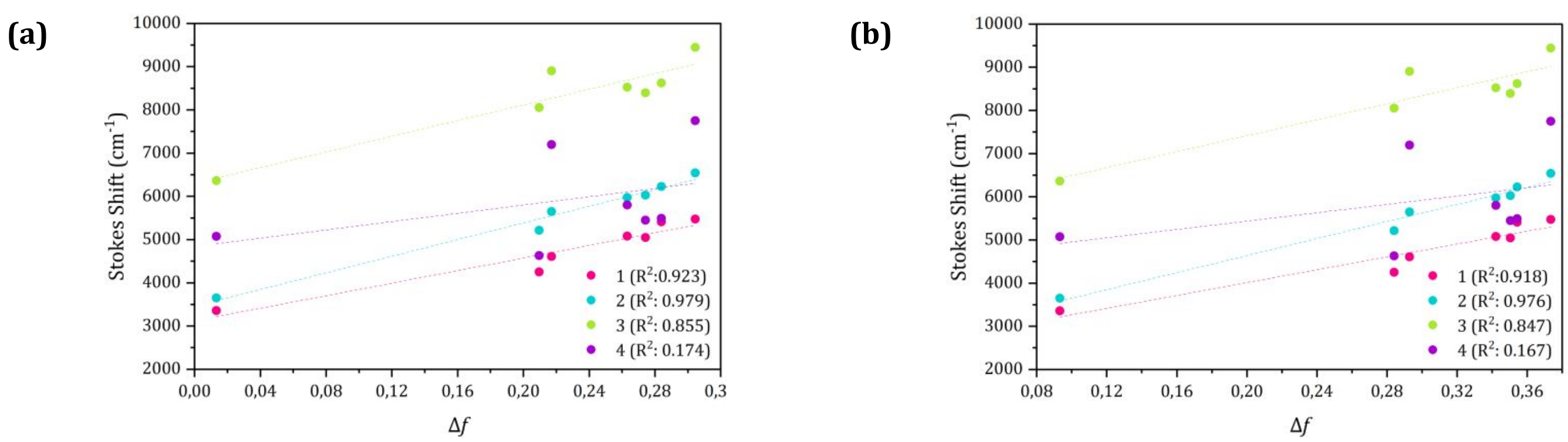

Figure S14. a) Lippert-Mataga and b) Rettig plots of compounds **1-4**.

Table S1. Computed E_{HOMO}, E_{LUMO} and the HOMO-LUMO gap (in eV) for compounds **1-4** from ground-state DFT in vacuum.

Compounds	E_{HOMO}	E_{LUMO}	HOMO-LUMO gap
**1**	-7.70	0.33	8.03
**2**	-7.51	0.079	7.59
**3**	-7.89	-0.11	7.78
**4**	-7.80	-0.43	7.37

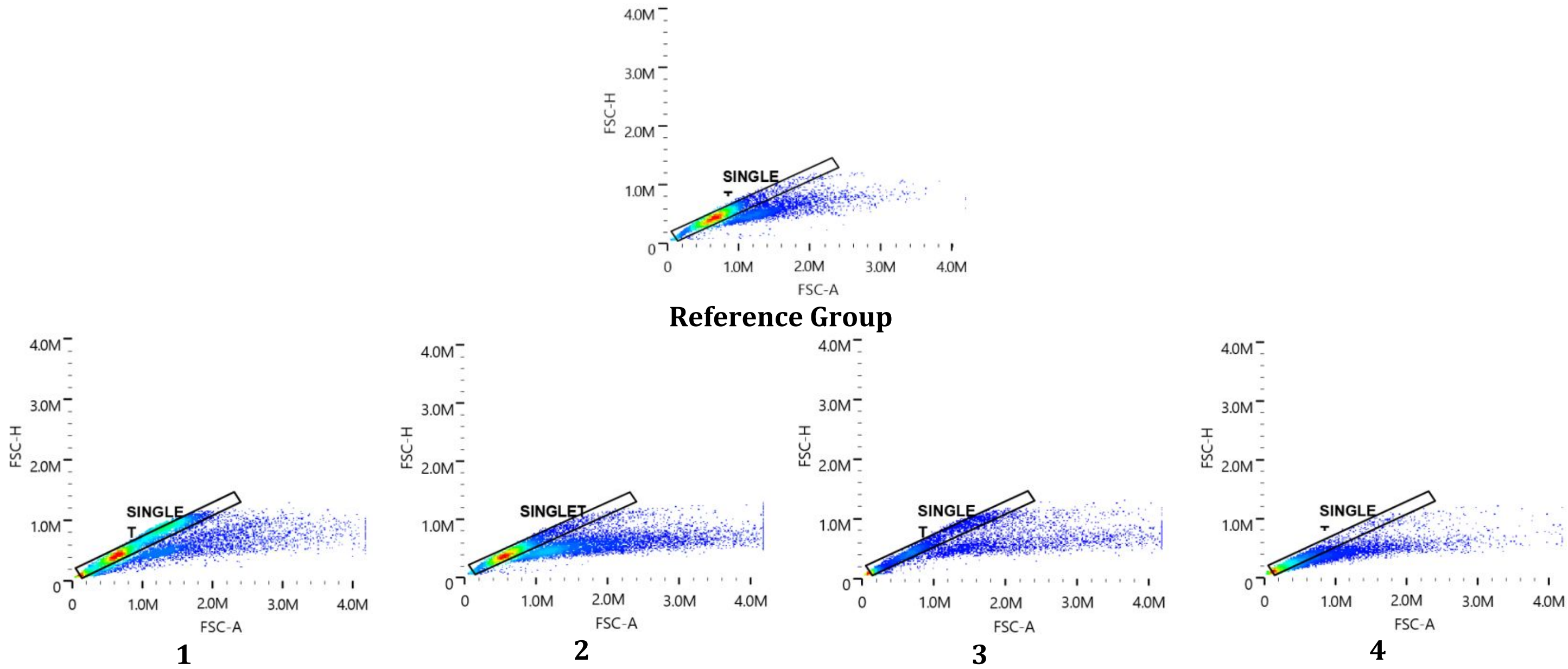

Figure S15. Morphological parameters (FSC and SSC) of compounds **1-4**.

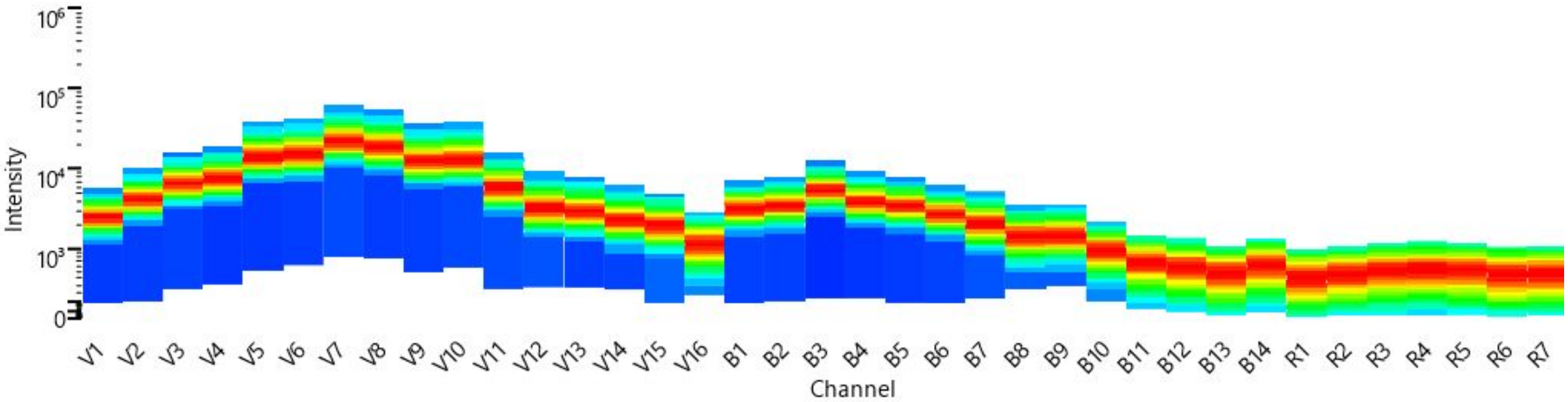

Reference Group SINGLET

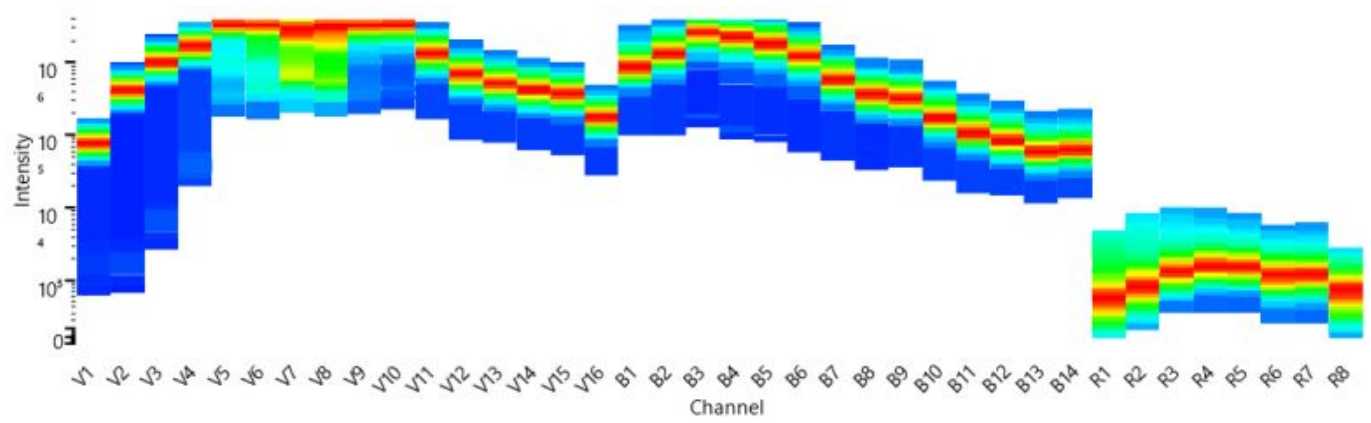

1 SINGLET

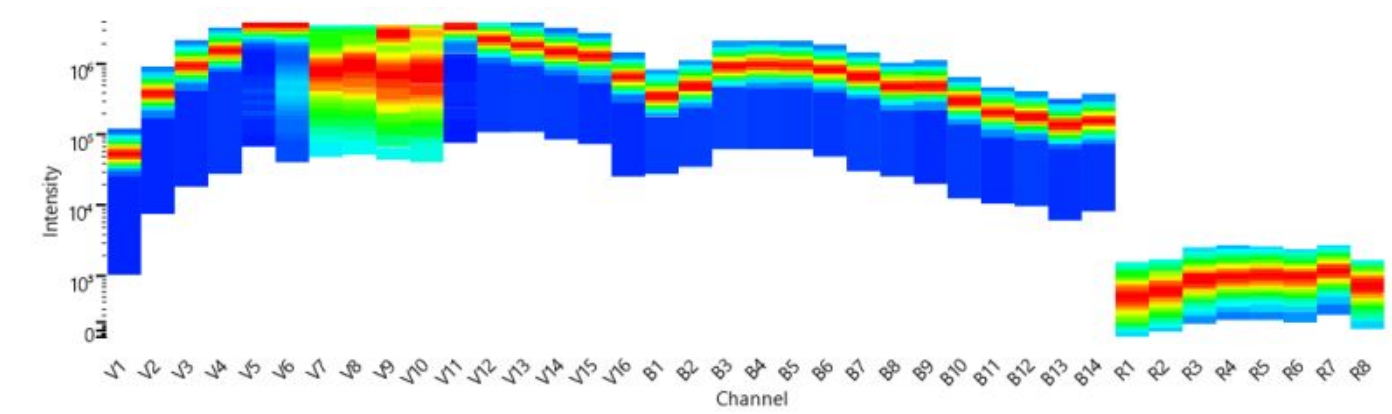

2 SINGLET

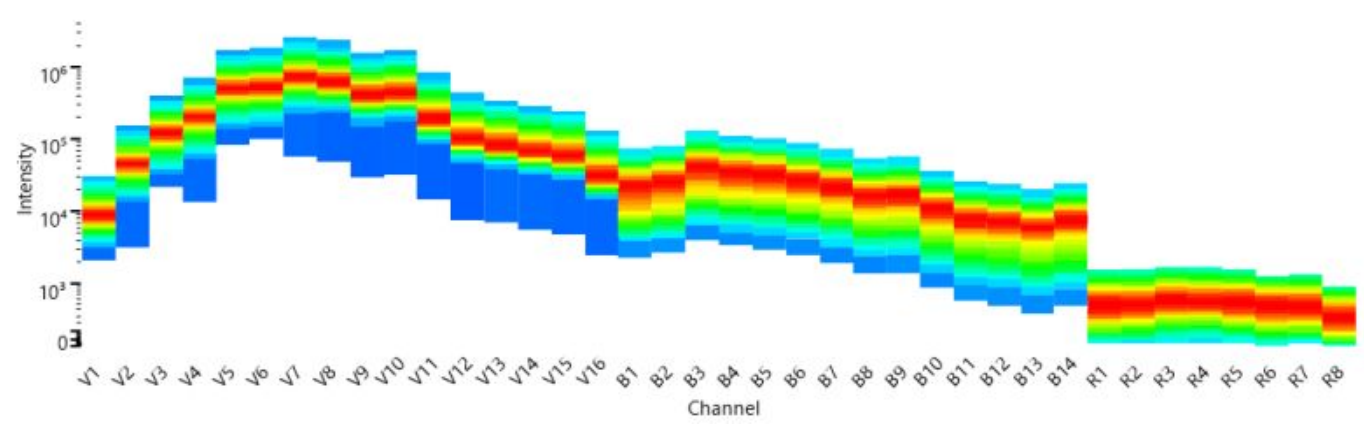

3 SINGLET

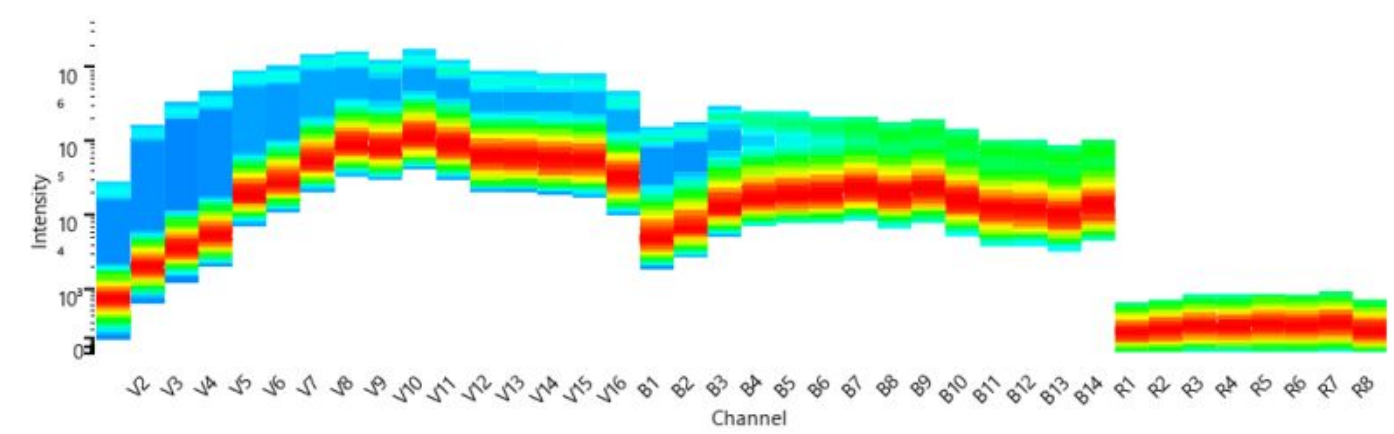

4 SINGLET

Figure S16. Flow cytometry of compounds **1-4.**
